# Effect of Chinese herbal medicine therapy on risks of all-cause mortality, infections, parasites, and circulatory-related mortality in HIV/AIDS patients with neurological diseases

**DOI:** 10.3389/fphar.2023.1097862

**Published:** 2023-03-03

**Authors:** Jian-Shiun Chiou, Chen-Hsing Chou, Mao-Wang Ho, Ni Tien, Wen-Miin Liang, Mu-Lin Chiu, Fuu-Jen Tsai, Yang-Chang Wu, I-Ching Chou, Hsing-Fang Lu, Ting-Hsu Lin, Chiu-Chu Liao, Shao-Mei Huang, Te-Mao Li, Ying-Ju Lin

**Affiliations:** ^1^ PhD Program for Health Science and Industry, College of Healthcare, China Medical University, Taichung, Taiwan; ^2^ Section of Infectious Diseases, Department of Internal Medicine, China Medical University Hospital, Taichung, Taiwan; ^3^ Department of Internal Medicine, School of Medicine, China Medical University, Taichung, Taiwan; ^4^ Department of Medical Laboratory Science and Biotechnology, China Medical University, Taichung, Taiwan; ^5^ Department of Health Services Administration, China Medical University, Taichung, Taiwan; ^6^ School of Chinese Medicine, China Medical University, Taichung, Taiwan; ^7^ Genetic Center, Department of Medical Research, China Medical University Hospital, Taichung, Taiwan; ^8^ Department of Biotechnology and Bioinformatics, Asia University, Taichung, Taiwan; ^9^ Department of Pediatrics, China Medical University Children’s Hospital, Taichung, Taiwan; ^10^ Graduate Institute of Integrated Medicine, China Medical University, Taichung, Taiwan

**Keywords:** HIV/AIDS, neurological diseases, mortality, Chinese herbal medicine, network analysis

## Abstract

**Introduction:** Long-term living with human immunodeficiency virus (HIV) and/or antiretroviral therapy (ART) is associated with various adverse effects, including neurocognitive impairment. Heterogeneous neurocognitive impairment remains an important issue, affecting between 15–65% of human immunodeficiency virus infection and acquired immunodeficiency syndrome (HIV/AIDS) patients and resulting in work performance, safety, and health-related outcomes that have a heavy economic burden.

**Methods:** We identified 1,209 HIV/AIDS patients with neurological diseases during 2010–2017. The Kaplan–Meier method, log-rank test, and Cox proportional hazards model were used to analyze 308 CHM users and 901 non-CHM users within this population. Major CHM clusters were determined using association rule mining and network analysis.

**Results and Discussion:** Results showed that CHM users had a 70% lower risk of all-cause mortality (adjusted hazard ratio (aHR) = 0.30, 95% confidence interval (CI):0.16–0.58, *p* < 0.001) (p = 0.0007, log-rank test). Furthermore, CHM users had an 86% lower risk of infections, parasites, and circulatory-related mortality (aHR = 0.14, 95% confidence interval (CI):0.04–0.46, *p* = 0.001) (*p* = 0.0010, log-rank test). Association rule mining and network analysis showed that two CHM clusters were important for patients with neurological diseases. In the first CHM cluster, Huang Qin (HQ; root of *Scutellaria baicalensis* Georgi), Gan Cao (GC; root of *Glycyrrhiza uralensis* Fisch.), Huang Lian (HL; root of *Coptis chinensis* Franch.), Jie Geng (JG; root of *Platycodon grandiflorus* (Jacq.) A.DC.), and Huang Bai (HB; bark of *Phellodendron amurense* Rupr.) were identified as important CHMs. Among them, the strongest connection strength was identified between the HL and HQ. In the second CHM cluster, Suan-Zao-Ren-Tang (SZRT) and Ye Jiao Teng (YJT; stem of *Polygonum multiflorum* Thunb.) were identified as important CHMs with the strongest connection strength. CHMs may thus be effective in treating HIV/AIDS patients with neurological diseases, and future clinical trials are essential for the prevention of neurological dysfunction in the population.

## 1 Introduction

Human immunodeficiency virus infection and acquired immunodeficiency syndrome (HIV/AIDS) is a chronic, yet manageable disease that is commonly treated using combinatorial antiretroviral therapy (ART), also known as highly active antiretroviral therapy (HAART) (Barbier et al., 2020). In the era of combinatorial ART, patients with HIV/AIDS have shown prolonged life expectancy, delayed disease progression, and lower all-cause mortality (Antiretroviral Therapy Cohort, 2017; Lu et al., 2018). The long-term use of ART and living with HIV/AIDS are associated with numerous adverse effects, including hyperlipidemia (Tsai F. J. et al., 2017), cardiovascular disease (Dorjee et al., 2017), loss of bone density (Hoy et al., 2017; Chiu et al., 2021b), and neurocognitive impairment (Yuan and Kaul, 2021).

Neurocognitive impairment includes central nervous system (CNS) infections, cognitive disorders, vasculopathy, and peripheral neuropathy (Tsai Y. T. et al., 2017), affecting patients in many ways, including intellectual dysfunction, poor memory and thinking skills, behavioral problems, and difficulty in performing daily activities (Gorman et al., 2009). The prevalence of neurocognitive impairment ranges between 15% and 65% owing to cohort characteristics and heterogeneous HIV-related neurocognitive diseases (Trujillo et al., 1995; Joska et al., 2011; Dai et al., 2014). The pathological mechanism between ART and/or HIV and neurocognitive impairment remains unclear, but it is probably due to the interactions between ART and HIV in the CNS (Brew et al., 1997; Wright et al., 2010; Shikuma et al., 2012; Sharma, 2021; Ruhanya et al., 2022; Wallace, 2022). Neurocognitive impairment is associated with HIV virus-induced neurotoxicity and immune suppression in the CNS (Brew et al., 1997; Ruhanya et al., 2022; Wallace, 2022) and is also associated with ART-related neurotoxicity (Sharma, 2021), persistent low-grade chronic inflammation, immune reactivation, low-level viral replication in the CNS, and aging-related comorbidities (Wright et al., 2010; Shikuma et al., 2012). Patients receiving ART with higher CPE scores are indicated to be at a higher risk of neurocognitive impairment (Marra et al., 2009; Caniglia et al., 2014).

Chinese herbal medicine (CHM) has been used in patients with HIV/AIDS-related diseases (Tsai F. J. et al., 2017; Tsai et al., 2018; Sun et al., 2019; Ho et al., 2021; Jiang et al., 2022). CHMs and related natural compounds have been reported to have anti-cognitive, anti-neuroinflammatory, and anti-HIV activities (Han et al., 2011; Cheng et al., 2015; Esposito et al., 2016; Zhao et al., 2016; Lee et al., 2017; Adebiyi et al., 2018; Long F. Y. et al., 2018; Zhang et al., 2018; Zeng et al., 2019; Qu et al., 2021; Dong et al., 2022; Xu et al., 2022; Xue et al., 2022). These studies encourage the investigation of whether CHM could improve survival in patients with neurological diseases HIV/AIDS as a complementary therapy to conventional medicine. Therefore, we evaluated the effect of CHM treatment on all-cause mortality and infections, parasites, and circulatory-related mortality in HIV/AIDS patients with neurological diseases in Taiwan using a population-based nationwide database.

## 2 Materials and methods

### 2.1 Study subjects

**FIGURE 1:**
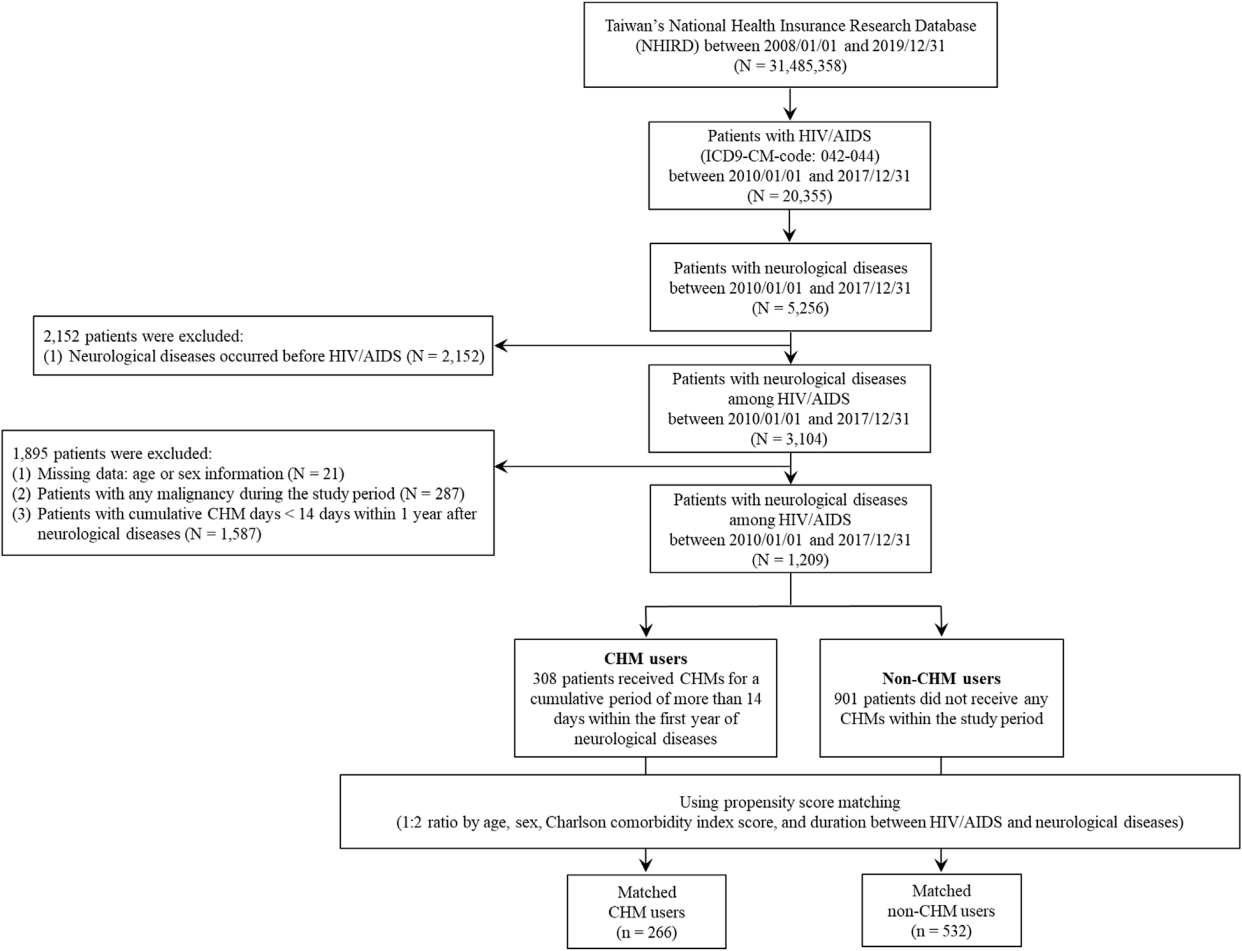
Flowchart for the enrollment of CHM and non-CHM users in HIV/AIDS patients with neurological diseases. Abbreviations: CHM, Chinese herbal medicine.

This longitudinal retrospective cohort study was conducted between 2008 and 2019 using the Taiwan National Health Insurance Research Database (NHIRD). Patients in the NHIRD were anonymized, and 20,355 patients with HIV/AIDS were identified with at least one inpatient or three outpatient visits within 1 year, as determined by the International Classification of Disease, 9th Revision, Clinical Modification (ICD-9-CM) codes for 042-044 between 2010 and 2017 (Figure 1). Patients were further classified based on whether they had neurological diseases also with at least one inpatient or three outpatient visits within 1 year: 1) CNS infections: the ICD-9-CM codes: 013, 047, 053, 094, 200, 320, 321, 322, 323, 054.3, 054.4, 114.2, 130.0, 321.0, 003.21, 098.82, 112.83, and 115.91; 2) cognitive disorders: the ICD-9-CM codes: 290, 293, 294, 332, 345, 348.1, 348.3, and 780.3; 3) vasculopathy: the ICD-9-CM codes: 325, 430, 431, 432, 433, 434, 435, 436, and 437; and 4) peripheral neuropathy: the ICD-9-CM codes: 350, 351, 353, 354, 355, 356, 357, and 358) (Tsai Y. T. et al., 2017). Of these categories, the following types of patients were excluded: 1) neurological diseases diagnosed prior to HIV/AIDS diagnosis (N = 2,152); 2) missing age or sex information (N = 21); 3) patients with <14 days’ cumulative prescription of CHM in 1 year after neurological disease diagnosis (N = 1,587); and 4) any malignancy during the study period (N = 287) (Figure 1). According to the frequency distribution of neurological disease subtypes among patients with HIV/AIDS, we observed that more than 75% patients belong to the CNS infections and cognitive disorders (Supplementary Figure S3). Also, for these patients, more than 75% of the inpatient and outpatient visits belongs to the clinics including Infectious Disease, International Medicine, Emergency Medicine, and Psychiatry (Supplementary Table S7).

**FIGURE 2:**
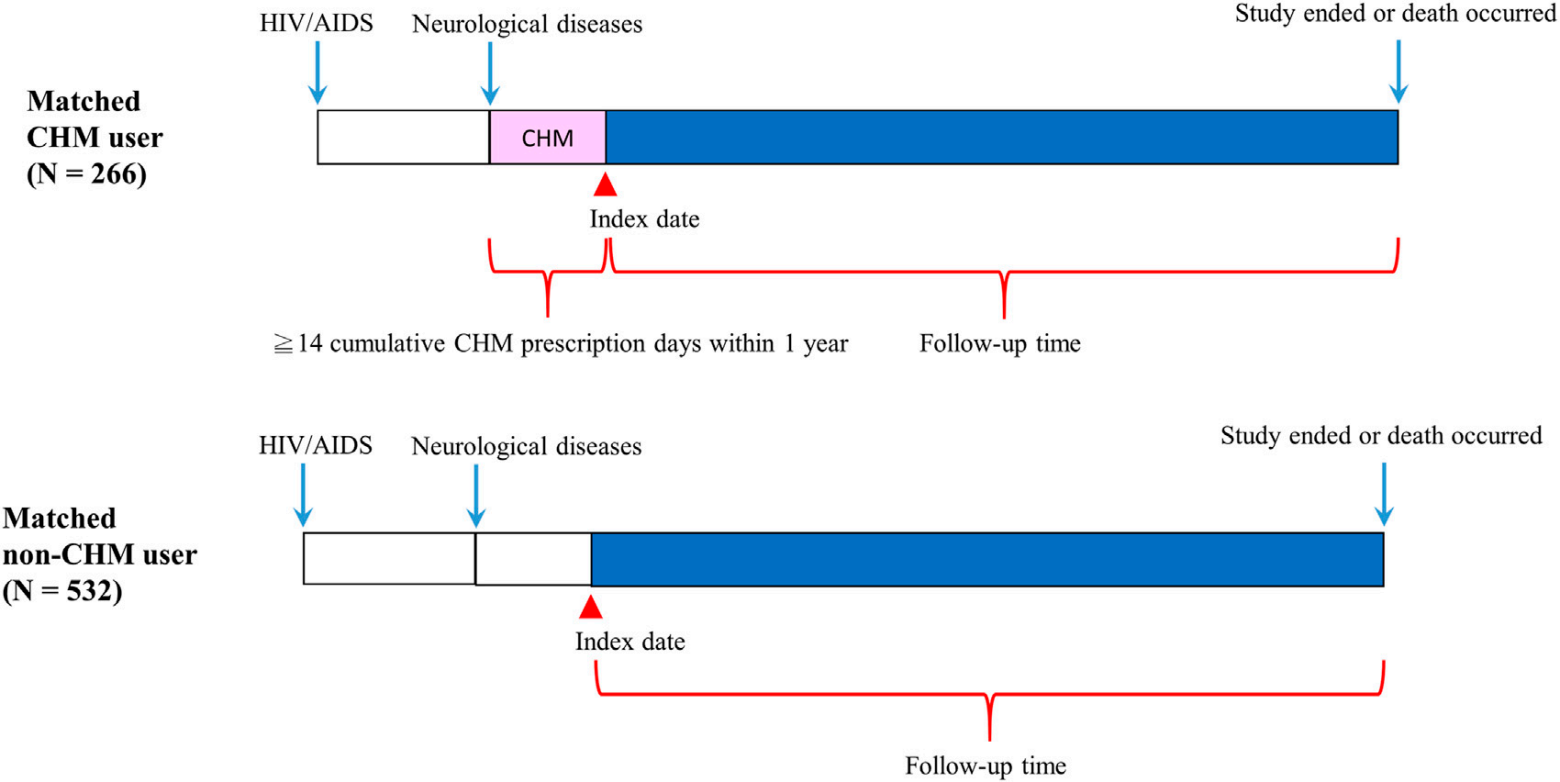
Follow-up times of CHM and non-CHM users in HIV/AIDS patients with neurological diseases. Abbreviations: CHM, Chinese herbal medicine.

After exclusion, a total of 1,209 patients with neurological diseases were included, including 901 non-CHM and 308 CHM users (Figure 1). Patients who received >14 days of cumulative CHM prescription in the first year after the onset of their neurological diseases were defined as CHM users (Figure 1). The date after the 14 days’ cumulative prescription of CHM was designated the index date. Non-CHM users were determined to be those without any CHM treatment during the study period (N = 901). Both groups were matched for sex, age, Charlson comorbidity index (CCI) score, and duration between HIV/AIDS and neurological diseases at a ratio of 1:2 using the propensity score matching method to reduce potential confounders (Table 1). Finally, 266 CHM and 532 non-CHM users were identified (Figures 1, 2; Table 1). Comorbidities were determined before HIV/AIDS diagnosis (Table 1). The cumulative defined daily doses (DDDs) of ART drugs were obtained during the index duration (Table 1). The index duration indicates the date of first diagnosis of HIV/AIDS and the date of first diagnosis of neurological diseases. The approval number (CMUH107-REC3-074(CR1)) was provided by China Medical University Hospital.

**TABLE 1:** Basic characteristics of patients with neurological diseases among HIV/AIDS according to Chinese herbal medicine usage in Taiwan.

Characteristics	Included subjects		*p*-value	Matched subjects		*p*-value
	CHM users (N = 308)	Non-CHM users (N = 901)		CHM users (N = 266)	Non-CHM users (N = 532)	
	N (%)	N (%)		N (%)	N (%)	
Age (years old)			***<0.001***			0.381
0≦Age<30	139 (45.13%)	263 (29.19%)		113 (42.48%)	217 (40.79%)	
30≦Age<40	107 (34.74%)	348 (38.62%)		93 (34.96%)	211 (39.66%)	
40≦Age	62 (20.13%)	290 (32.19%)		60 (22.56%)	104 (19.55%)	
Sex			0.487			0.898
Male	295 (95.78%)	854 (94.78%)		255 (95.86%)	511 (96.05%)	
Female	13 (4.22%)	47 (5.22%)		11 (4.14%)	21 (3.95%)	
Index duration (day; Mean ± SD)	761.22 ± 789.83	818.29 ± 874.95	0.288	786.40 ± 804.10	766.60 ± 851.18	0.752
Cumulative DDDs of ART drugs (Mean ± SD)	1,691.77 ± 2,219.84	1,451.29 ± 1969.10	0.164	1766.71 ± 2,297.08	1,405.38 ± 2000.16	0.070
NRTI cumulative DDDs (Mean ± SD)	733.82 ± 996.39	776.45 ± 1,106.21	0.796	769.67 ± 1,026.07	826.12 ± 1,294.86	0.789
PI cumulative DDDs (Mean ± SD)	805.42 ± 1,027.59	776.85 ± 1,040.04	0.888	791.61 ± 994.86	771.62 ± 1,087.37	0.931
NNRTI cumulative DDDs (Mean ± SD)	396.89 ± 514.53	336.5 ± 524.43	0.221	403.31 ± 521.27	276.34 ± 447.55	***0.017***
INSTI cumulative DDDs (Mean ± SD)	682 ± 962.5	384.42 ± 524.81	0.139	703.62 ± 974.85	343.53 ± 371.17	0.084
Combined ART cumulative DDDs (Mean ± SD)	1,049.84 ± 1,613.64	877.79 ± 1,307.75	0.176	1,095.83 ± 1,695.64	904.36 ± 1,367.61	0.199
Charlson comorbidity index score (CCI score; Mean ± SD)	0.41 ± 0.77	0.31 ± 0.71	***0.032***	0.32 ± 0.67	0.27 ± 0.62	0.238
Charlson comorbidity number			0.052			0.302
0	220 (71.43%)	704 (78.14%)		202 (75.94%)	428 (80.45%)	
1-2	66 (21.43%)	152 (16.87%)		50 (18.80%)	78 (14.66%)	
≥3	22 (7.14%)	45 (4.99%)		14 (5.26%)	26 (4.89%)	
Opportunistic infections						
Pneumocystis jirovecii pneumonia			0.127			0.172
No	276 (89.61%)	777 (86.24%)		239 (89.85%)	460 (86.47%)	
Yes	32 (10.39%)	124 (13.76%)		27 (10.15%)	72 (13.53%)	
Cytomegalovirus disease (other than liver, spleen, or nodes)			0.628			0.451
No	296 (96.1%)	860 (95.45%)		257 (96.62%)	508 (95.49%)	
Yes	12 (3.9%)	41 (4.55%)		9 (3.38%)	24 (4.51%)	
*Mycobacterium tuberculosis*			0.227			0.231
No	300 (97.4%)	864 (95.89%)		261 (98.12%)	514 (96.62%)	
Yes	8 (2.6%)	37 (4.11%)		5 (1.88%)	18 (3.38%)	
Candidiasis (esophagus, bronchi, trachea, lung)			0.123			0.348
No	303 (98.38%)	871 (96.67%)		261 (98.12%)	516 (96.99%)	
Yes	5 (1.62%)	30 (3.33%)		5 (1.88%)	16 (3.01%)	
Cryptococcosis, extrapulmonary			0.543			0.625
No	294 (95.45%)	852 (94.56%)		253 (95.11%)	510 (95.86%)	
Yes	14 (4.55%)	49 (5.44%)		13 (4.89%)	22 (4.14%)	
Disseminated *Mycobacterium avium* complex infection or M. kansasii			0.582			0.323
No	301 (97.73%)	885 (98.22%)		260 (97.74%)	525 (98.68%)	
Yes	7 (2.27%)	16 (1.78%)		6 (2.26%)	7 (1.32%)	

N, number; ART, antiretroviral therapies; cumulative DDDs, cumulative defined daily doses; CHM, chinese herbal medicine; SD, standard deviation; CCI, charlson comorbidity index; NRTI, nucleoside reverse-transcriptase inhibitor; PI, protease inhibitor; NNRTI, non-nucleoside reverse transcriptase inhibitor; INSTI, integrase strand transfer inhibitor.

*p*-values for age, sex, and Charlson comorbidity number were obtained by the chi-square test; *p*-values for index duration, cumulative DDDs, of ART, drugs, and Charlson comorbidity index score were obtained by the un-paired Student’s t-test.

Significant *p*-values (*p* < 0.05) are highlighted in bold italic font.

Index duration was from the diagnosed date of HIV/AIDS, to the diagnosed date of neurological diseases (day; Mean ± SD). Cumulative DDDs, of ART, drugs were obtained during the index duration.

Pneumocystis jirovecii pneumonia: the ICD-9-CM, code: 136.3 and the ICD-10-CM, code: B59; cytomegalovirus disease (other than liver, spleen, or nodes): the ICD-9-CM, codes: 078.5 and 484.1 and the ICD-10-CM, codes: B25.0, B25.2, and B25.8; *mycobacterium tuberculosis*: the ICD-9-CM, codes: 010–012 and 018 and the ICD-10-CM, codes: A15.X-A19.X; candidiasis (esophagus, bronchi, trachea, lung): the ICD-9-CM, codes: 112.4 and 112.84 and the ICD-10-CM, codes: B37.1 and B37.81; cryptococcosis, extrapulmonary: the ICD-9-CM, codes: 117.5 and 321.0 and the ICD-10-CM, codes: B45.1-B45.8; Disseminated *Mycobacterium avium* complex infection or M. kansasii: the ICD-9-CM, codes: 031.2 and 031.9 and the ICD-10-CM, codes: A31.2 and A31.9.

### 2.2 Prescription patterns, network analysis, and association rule mining for CHM

Traditional Chinese medicine (TCM) is part of the regular healthcare system in Taiwan. In this study, CHMs were produced by Good Manufacturing Practice pharmaceutical companies and used by traditional Chinese medicine doctors for treatment. CHMs have two forms, i.e., single herb and herbal formula. The leaf, stem, root, or flower of a plant, as well as the organ of an insect, animal, or mineral source, could be used as a single herb, while herbal formulas comprise more than two single herbs. The CHM prescription pattern is shown in Supplementary Table S1 for Taiwanese HIV/AIDS patients with neurological diseases.

Association rule mining was conducted as previously described (Wang et al., 2020; Wu et al., 2020; Chiu et al., 2021a; Chen et al., 2021; Chou et al., 2021; Ho et al., 2021; Chiu et al., 2022), using the SAS software (version 9.4; SAS Institute, Cary, NC, United States). The connection strength between the two types of CHMs was calculated using lift values, confidence, and support for the co-prescription of CHM_X and CHM_Y (Table 4). The lift value is confidence (CHM_X → CHM_Y) (%)/*p* (Y) (%) or confidence (CHM_X → CHM_Y) (%)/*p* (X) (%). The lift value is the ratio of the observed support to the expected support, when X and Y are independent variables. A lift value greater than one suggests a strong association between CHM_X and CHM_Y, indicating that the association between the two CHMs is dependent. The confidence value is the conditional probability of receiving CHM_Y among those who already received CHM_X, which is calculated as follows (frequency of CHM_X and CHM_Y/frequency of CHM_X) × 100%. The confidence value (CHM_X → CHM_Y; %) is an indicator of how often CHM_Y appeared in calculations that contained CHM_X. Support value is the joint possibility of receiving both CHM_X and CHM_Y, which is calculated by (frequency of CHM_X and CHM_Y/total number of prescriptions) × 100%. Support value (X, %) is a measure of whether an association between CHM_X and CHM_Y occurred by chance.

Network analysis was performed as described previously (Wang et al., 2020; Wu et al., 2020; Chiu et al., 2021a; Chen et al., 2021; Chou et al., 2021; Ho et al., 2021; Chiu et al., 2022) (Figure 4). The green circle indicates a single herb, while the red circle indicates a herbal formula. A larger circle size was associated with a higher prescription frequency of CHM (Supplementary Tables S1, S2), and line size and color between CHM_X and CHM_Y represent the connection strength. The thicker line shows a higher support value between the CHMs (Table 4), while a darker line indicates a higher lift value (Table 4). Cytoscape software was used to analyze all the data (https://cytoscape.org/, version 3.7.0).

### 2.3 Statistical analysis

**FIGURE 3:**
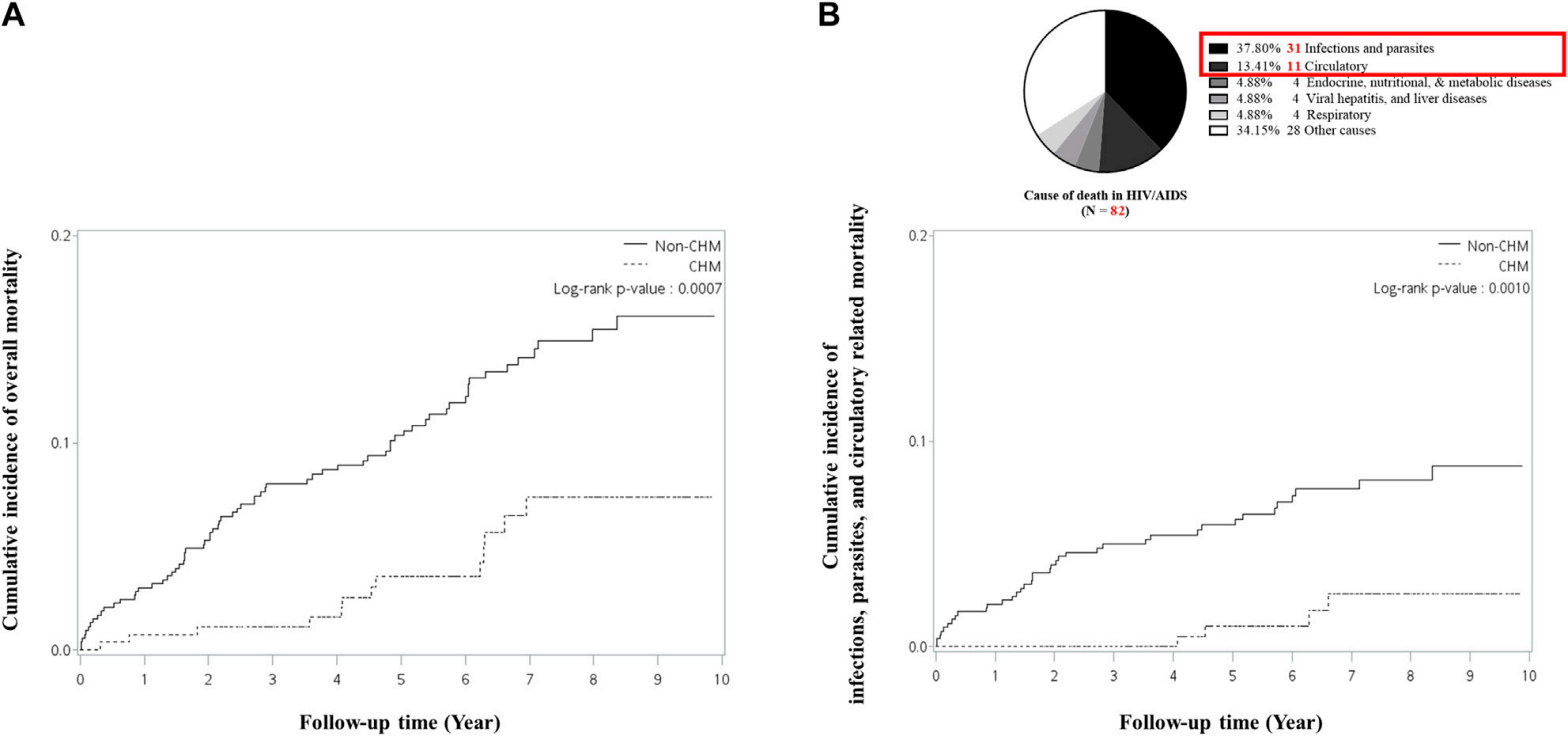
Cumulative incidence of **(A)** all-cause mortality and **(B)** infections, parasites, and circulatory-related mortality between CHM and non-CHM users in HIV/AIDS patients with neurological diseases. Abbreviations: CHM, Chinese herbal medicine.

Categorical data, including age, sex, and Charlson comorbidity numbers, are presented as numbers (percentages) (Table 1). Categorical and continuous data were calculated using the chi-square test and unpaired Student’s t-test, respectively. The CCI score, cumulative DDDs of ART drugs, and index duration were presented as continuous data. The risks of all-cause mortality and infections, parasites, and circulatory-related mortality were estimated using univariate and multivariate Cox proportional hazard models (Tables 2, 3, Supplementary Tables S3, S5, S6, and S10). Adjusted factors included sex, age, Charlson comorbidity, and CHM use. The cumulative incidence of mortality between CHM and non-CHM users was estimated using the Kaplan-Meier method and log-rank test (Figure 3; Supplementary Figures S1, S2). SAS analysis was performed using the statistical software (version 9.4; SAS Institute, Cary, NC, United States).

**TABLE 2:** Cox proportional hazard models for risk of all-cause mortality in patients with neurological diseases among HIV/AIDS.

	All-cause mortality					
	Crude			Adjusted		
	HR	95% CI	*p*-value	aHR	95% CI	*p*-value
Age (years old)						
30 ≦ Age < 40 (vs. 0≦ Age <30)	1.49	(0.83–2.68)	0.185	1.26	(0.68–2.32)	0.461
40 ≦ Age (vs. 0≦ Age <30)	4.48	(2.64–7.60)	***<0.001***	3.79	(2.12–6.76)	***<0.001***
Female (vs. male)	2.98	(1.40–6.32)	***0.005***	2.80	(1.24–6.34)	***0.013***
CHM use (vs. non-CHM use)	0.38	(0.21–0.66)	***<0.001***	0.30	(0.16–0.58)	***<0.001***
Charlson comorbidity number_1-2 (vs. 0)	1.80	(1.05–3.10)	***0.033***	1.38	(0.77–2.47)	0.284
Charlson comorbidity number_≥3 (vs. 0)	3.90	(2.00–7.64)	***<0.001***	1.91	(0.90–4.04)	0.092

HR, hazard ratio; aHR, adjusted hazard ratio; CI, confidence interval; CHM, Chinese herbal medicine. Adjusted factors: age, sex, CHM, use, and Charlson comorbidity number. Significant *p*-values (*p* < 0.05) are highlighted in bold italic font.

**TABLE 3:** Cox proportional hazard models for risk of infections, parasites, and circulatory-related mortality in patients with neurological diseases among HIV/AIDS.

	Infections, parasites, and circulatory-related mortality					
	Crude			Adjusted		
	HR	95% CI	*p*-value	aHR	95% CI	*p*-value
Age (years old)						
30 ≦ Age < 40 (vs. 0 ≦ Age <30)	1.55	(0.60–4.00)	0.361	1.24	(0.47–3.29)	0.664
40 ≦ Age (vs. 0 ≦ Age <30)	8.09	(3.57–18.32)	***<0.001***	6.57	(2.79–15.46)	***<0.001***
Female (vs. male)	3.69	(1.42–9.57)	***0.007***	3.80	(1.34–10.76)	***0.012***
CHM use (vs. non-CHM use)	0.21	(0.08–0.58)	***0.003***	0.14	(0.04–0.46)	***0.001***
Charlson comorbidity number_1-2 (vs. 0)	2.19	(1.05–4.59)	***0.037***	1.49	(0.69–3.20)	0.312
Charlson comorbidity number_≥3 (vs. 0)	6.13	(2.59–14.54)	***<0.001***	2.36	(0.92–6.02)	0.073

HR, hazard ratio; aHR, adjusted hazard ratio; CI, confidence interval; CHM, chinese herbal medicine.

Adjusted factors: age, sex, CHM, use, and Charlson comorbidity number.

Significant *p*-values (*p* < 0.05) are highlighted in bold italic font.

## 3 Results

### 3.1 Basic characteristics

The basic characteristics of patients with neurological diseases among the HIV/AIDS patients in Taiwan are presented (Table 1). For the subjects in this study, 308 CHM users and 901 non-CHM users were included. CCI score and age were significantly different between CHM and non-CHM users (*p* < 0.05; Table 1). To avoid confounding effects, propensity score matching was used to match the two groups at a 1:2 ratio for the duration between HIV/AIDS and neurological diseases, CCI score, sex, and age. After matching, there were no background differences between the 266 CHM users and 532 non-CHM users (matched subjects) (*p* > 0.05; Table 1). The CHM users received CHM therapies during the study period (Supplementary Table S4). There were 266 CHM users who were treated with CHMs and 532 patients who were not treated with any CHM during the study period. Furthermore, a separate percentage of malignancies in the two groups was shown in Supplementary Table S8. As shown there was no reported HIV/AIDS related malignancy including Kaposi’s sarcoma, lymphoma, and invasive cervical cancer during the study period (*p* > 0.05).

### 3.2 All-cause mortality

The cumulative incidence of all-cause mortality between CHM and non-CHM users was estimated using the Kaplan–Meier survival model (Figure 3A). The cumulative incidence of all-cause mortality between CHM and non-users was significantly different (*p* = 0.0007). Compared with non-CHM users, CHM users showed a significantly decreased cumulative incidence of all-cause mortality.

For HIV/AIDS patients with neurological diseases, the risk of all-cause mortality was estimated using univariate and multivariate Cox proportional hazards models (Table 2). The crude hazard ratios (cHR) showed differences in sex, age, comorbidities, and CHM use (*p* < 0.05). Patients aged >40 years were found to be at a higher risk of all-cause mortality than those aged <30 years (crude hazard ratio (cHR):4.48, 95% confidence interval (CI): 2.64–7.60, *p* < 0.001). Females were at a higher risk of all-cause mortality than males (cHR, 2.98; 95% CI: 1.40–6.32, *p* = 0.005). CHM users had a lower risk of all-cause mortality than non-CHM users (cHR: 0.38, 95% CI: 0.21–0.66, *p* < 0.001). Patients with > three Charlson comorbidities were at a higher risk of all-cause mortality than those without any comorbidities (cHR: 3.90, 95% CI: 2.00–7.64, *p* < 0.001). Patients with one or two Charlson comorbidities were at a higher risk of all-cause mortality than those with no comorbidities (cHR, 1.80; 95% CI: 1.05–3.10, *p* = 0.033).

The adjusted hazard ratios (aHR) also showed differences in sex, age, and CHM use (*p* < 0.05) (Table 2). CCI score, CHM use, sex, and age were adjusted for in this model. Patients aged >40 years had a greater risk of all-cause mortality than those aged <30 years (adjusted HR (aHR), 3.79; 95% CI: 2.12–6.76, *p* < 0.001). Females had a greater risk of all-cause mortality than males (aHR: 2.80, 95% CI: 1.24–6.34, *p* = 0.013). CHM users had a lower risk of all-cause mortality than non-CHM users (aHR, 0.30; 95% CI: 0.16–0.58, *p* < 0.001). Furthermore, we observed that CHM users still had a lower risk of all-cause mortality than non-CHM users after considering the adjusted factors, age, sex, Charlson comorbidity number, and the interval (between the diagnostic date of neurological diseases and the index date) (adjusted HR:0.31, 95% CI: 0.16–0.59, *p* < 0.001) (Supplementary Table S3). Frequency of CHM use was associated with a reduced risk of overall mortality in patients with neurological diseases among HIV/AIDS (Supplementary Figures S1, S2) (Supplementary Tables S5, S6). For the sensitivity test, we observed that CHM users still had a lower risk of all-cause mortality than non-CHM users in patients with the CNS infections (Supplementary Figure S4: log rank *p* = 0.0009) (Supplementary Table S10: adjusted HR: 0.10, 95% CI: 0.03–0.39, *p* < 0.001).

### 3.3 Infections, parasites, and circulatory system-related mortality

In addition to all-cause mortality, we classified deaths according to the cause of death information into categories using ICD-9-CM and ICD-10-CM codes (Eyawo et al., 2017; Long L. C. et al., 2018). In this study, the causes of death were grouped into the following categories: infections and parasites, circulatory, endocrine, nutritional, and metabolic diseases, viral hepatitis, and liver diseases, respiratory, genitourinary system, neoplasms, and other causes, which includes all causes of death not listed in the above mentioned categories (Supplementary Table S9). Supplementary Table S9 lists the ICD-9-CM and ICD-10-CM codes associated with the causes of death categories. Among the causes of death in HIV/AIDS patients with neurological diseases, approximately 51% of patients had infections, parasites, and circulatory-related mortality (Figure 3B) (Supplementary Table S9). Patients with other causes of mortality were excluded from the final analysis when evaluating infections, parasites, and circulatory-related mortality.

The cumulative incidence of infections, parasites, and circulatory-related mortality between CHM and non-CHM users was calculated using a Kaplan–Meier survival plot (Figure 3B). The cumulative incidences of infections, parasites, and circulatory-related mortality between CHM and non-users were significantly different (*p* = 0.0010). Compared to non-CHM users, CHM users had a significantly decreased cumulative incidence of infections, parasites, and circulatory-related mortality.

The cHR and aHR were calculated for the risk of infections, parasites, and circulatory-related mortality (Table 3). cHRs showed differences in comorbidities, CHM use, sex, and age (*p* < 0.05). Patients aged >40 years were at a greater risk than those aged <30 years (cHR:8.09, 95% confidence interval (CI): 3.57–18.32, *p* < 0.001). Females were at a greater risk than males (cHR: 3.69, 95% CI: 1.42–9.57, *p* = 0.007). CHM users were at a lower risk than non-CHM users (cHR: 0.21, 95% CI: 0.08–0.58, *p* = 0.003). Patients with more than three Charlson comorbidities were at a greater risk than those without any comorbidities (cHR, 6.13; 95% CI: 2.59–14.54, *p* < 0.001). Patients with one or two Charlson comorbidities were at a greater risk than those with no comorbidities (cHR: 2.19, 95% CI: 1.05–4.59, *p* = 0.037).

The aHRs showed differences in CHM use, sex, and age (*p* < 0.05) (Table 3). CCI score, CHM use, sex, and age were adjusted for in this model. Patients aged >40 years had a greater risk than those aged <30 years (aHR: 6.57, 95% CI: 2.79–15.46, *p* < 0.001). Females were at a greater risk than males (aHR: 3.80, 95% CI: 1.34–10.76, *p* = 0.012). CHM users had a lower risk than non-CHM users (aHR, 0.14; 95% CI: 0.04–0.46, *p* = 0.001).

### 3.4 CHM prescription pattern

According to prescription frequency, the most frequently used herbal formulas and single herbs were found in patients with neurological diseases among the HIV/AIDS patients in Taiwan (Supplementary Table S1). Among herbal formulas, Long-Dan-Xie-Gan-Tang (LDXGT) was the most commonly used herbal formula (prescription frequency: 459). The second, third, and fourth formulas were Ban-Xia-Xie-Xin-Tang (BXXXT) (prescription frequency: 407), Ge-Gen-Tang (GGT) (prescription frequency: 390), and Suan-Zao-Ren-Tang (SZRT) (prescription frequency: 280), respectively. The most frequently used single herb was Huang Qin (HQ; root of *Scutellaria baicalensis* Georgi) (prescription frequency: 557), followed by Gan Cao (GC; root of *Glycyrrhiza uralensis* Fisch.) (prescription frequency: 540), Da Huang (root and rhizome of DaH; *Rheum palmatum* L.) (prescription frequency: 528), and Jie Geng (JG; root of *Platycodon grandiflorus* (Jacq.) A.DC.) (prescription frequency: 466).

Association rule analysis was applied to explore the strongest associated CHM pairs for HIV/AIDS patients with neurological diseases (Table 4). According to prescription frequency, support, and lift values of CHM pairs, the most commonly used co-prescriptions of CHM pairs were listed: Huang Lian (HL; root of *Coptis chinensis* Franch.) → Huang Qin (HQ; root of *S. baicalensis* Georgi) (first co-occurrence frequency:145, support: 1.97%, confidence: 36.34%, lift: 4.81), followed by Suan-Zao-Ren-Tang (SZRT) → Ye Jiao Teng (YJT; stem of *Polygonum multiflorum* Thunb.) (second co-occurrence frequency: 96, support: 1.30%, confidence: 34.29%, lift: 7.81), and Gan Cao (GC; root of *G. uralensis* Fisch.) → Huang Qin (HQ; root of *S. baicalensis* Georgi) (third co-occurrence frequency: 91, support: 1.23%, confidence: 16.85%, lift: 2.23).

**TABLE 4:** Top five most commonly used co-prescriptions of CHM products for patients with neurological diseases among HIV/AIDS in Taiwan.

CHM products (LHS, X)	Chinese name	Frequency of prescriptions of X product	Dosage of X product		CHM products (RHS, Y)	Chinese name	Frequency of prescriptions of Y product	Dosage of Y product	Frequency of prescriptions of X and Y products	Support (X) (%)	Confidence (X → Y) (%)	Lift
Huang Lian (HL)	黃連	399	2,575	→	Huang Qin (HQ)	黃芩	557	6,917	145	1.97	36.34	4.81
Suan-Zao-Ren-Tang (SZRT)	酸棗仁湯	280	23,189	→	Ye Jiao Teng (YJT)	夜交藤	324	2,161	96	1.30	34.29	7.81
Gan Cao (GC)	甘草	540	3,938	→	Huang Qin (HQ)	黃芩	557	6,917	91	1.23	16.85	2.23
Jie Geng (JG)	桔梗	466	3,294	→	Gan Cao (GC)	甘草	540	3,938	91	1.23	19.53	2.67
Huang Bai (HB)	黃柏	280	1969	→	Huang Lian (HL)	黃連	399	2,575	88	1.19	31.43	5.81

CHM, chinese herbal medicine; LHS, left-hand-side; RHS, right-hand-side.

Total prescriptions = 7376.

Dosage of X or Y products = Average drug dose per day (g) × Average duration for prescription (days) × Frequency of prescriptions (Supplementary Table S1).

Support (X) (%) = Frequency of prescriptions of X and Y products/total prescriptions × 100%.

Confidence (X → Y) (%) = Frequency of prescriptions of X and Y products/Frequency of prescriptions of X product × 100%.

*p* (Y) (%) = Frequency of prescriptions of Y product/total prescriptions × 100%.

Lift = Confidence (X → Y) (%)/*p* (Y) (%).

**FIGURE 4:**
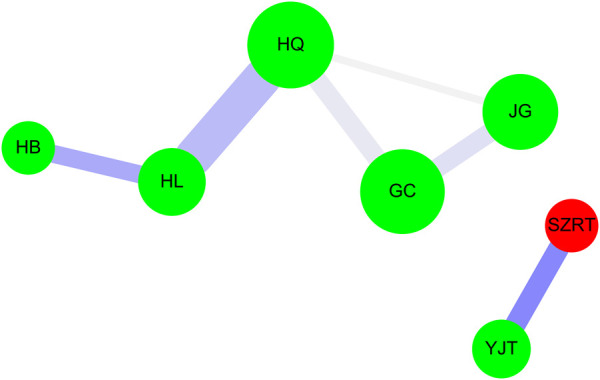
Network analysis for CHM prescription pattern in HIV/AIDS patients with neurological diseases. Single herb is presented as a green circle; herbal formula is displayed as a red circle. Bigger circle size shows the higher prescription frequency of CHM. The connection strength is shown as the line size and line color between CHM_X and CHM_Y. Thicker line represents the higher support value between CHMs. Darker line shows higher lift value. Abbreviations: CHM, Chinese herbal medicine; SZRT, Suan-Zao-Ren-Tang; YJT, Ye Jiao Teng; HQ, Huang Qin; HL, Huang Lian; GC: Gan Cao; JG: Jie Geng; HB: Huang Bai.

The CHM pairs described above were used to construct network analysis using the Cytoscape software (Figure 4). In this study, 266 patients received 7,376 CHM prescriptions during the study period (Table 4). Among HIV/AIDS patients, two CHM clusters were important for patients with neurological diseases.

In the first CHM cluster, HQ (root of *S. baicalensis* Georgi) and JG (root of *P. grandiflorus* (Jacq.) A.DC.), HL (root of *C. chinensis* Franch.), Huang Bai (HB; bark of Phellodendron amurense *Rupr.*), and GC (root of *G. uralensis* Fisch.) were important CHMs. Among this cluster, the core CHMs were identified as the HL (root of *C. chinensis* Franch.), and HQ (root of *S. baicalensis* Georgi) due to the most commonly used CHM pair with the strongest connection strength within this cluster. According to the Taiwan Herbal Pharmacopeia (4th Edition English Version, the Ministry of Health and Welfare, Taiwan) (https://dep.mohw.gov.tw/docmap/lp-759-108.html), the CHM effects of HL and HQ were to clear heat and dry dampness, purge fire and detoxicate, and induce diuresis to alleviate edema. Furthermore, the SymMap website (http://www.symmap.org/) was also used to explore the relationship among single herbs, modern medicine symptoms, and neurological diseases (Supplementary Figure S5). As shown in Supplementary Figure S5, in the first CHM cluster, HQ and HL targeted more modern medicine symptoms that belongs to these neurological diseases.

In the second CHM cluster, SZRT and YJT (stem of *P. multiflorum* Thunb.) were important CHMs with the strongest connection strength. Therefore, in this cluster, the core CHMs were SZRT and YJT due to the most commonly used CHM pair with the strongest connection strength within this cluster. Also, according to the Taiwan Herbal Pharmacopeia (4th Edition English Version, the Ministry of Health and Welfare, Taiwan) (https://dep.mohw.gov.tw/docmap/lp-759-108.html), the CHM effects of SZRT and YJT were to nourish the heart to tranquilize, relieve sweating, generate fluid, and to nourish the heart to tranquilize, dispel wind to free collateral vessels. Furthermore, the SymMap website (http://www.symmap.org/) was also used to explore the relationship among single herbs, modern medicine symptoms, and neurological diseases (Supplementary Figure S6). As shown in Supplementary Figure S6, in the second CHM cluster, both of SZRT and YJT targeted more modern medicine symptoms that belongs to these neurological diseases.

## 4 Discussion

Neurocognitive impairment remains an important issue in HIV/AIDS patients, even in the era of combinatorial ART (Mothobi and Brew, 2012; Yuan and Kaul, 2021). We evaluated the effectiveness of CHMs on the risk of mortality in HIV/AIDS patients with neurological diseases in Taiwan. We found that CHM users had a decreased risk of mortality, including all-cause mortality, infection, parasites, and circulatory-related mortalities. Frequency of CHM use was associated with a reduced risk of overall mortality in patients with neurological diseases among HIV/AIDS. We observed that CHM users still had a lower risk of all-cause mortality than non-CHM users in patients with the CNS infections. Two CHM clusters were identified. The first CHM cluster, HQ (root of *S. baicalensis* Georgi), JG (root of *P. grandiflorus* (Jacq.) A.DC.), HB (bark of *P. amurense* Rupr.), HL (root of *C. chinensis* Franch.), and GC (root of *G. uralensis* Fisch.) were important CHMs. In the second CHM cluster, SZRT and YJT (stem of *P. multiflorum* Thunb.) were found to be important CHMs. This study suggests that CHM use shows lower risks of all-cause mortality and infections, parasites, and circulatory-related mortality in patients with neurological diseases among HIV/AIDS patients in Taiwan.

HIV infection impairs the immune system and increases the risk of opportunistic infections in infected individuals (Luo et al., 2016; Gangcuangco et al., 2017; Lee et al., 2018). Opportunistic infections also contribute significantly to increased morbidity and mortality among these patients (Xiao et al., 2013; Chanto and Kiertiburanakul, 2020). The introduction of Combination antiretroviral therapy (ART) has led to a significant decrease in the occurrence of opportunistic infections among these patients (Palella et al., 2006; Mirani et al., 2015). Furthermore, studies have shown that 15%–65% of HIV/AIDS patients suffer from neurocognitive impairment (Trujillo et al., 1995; Joska et al., 2011; Dai et al., 2014). HIV virus-induced neurotoxicity, immune suppression in the CNS, ART-related neurotoxicity, or persistent low-grade chronic inflammation may lead to neurological impairment (Brew et al., 1997; Sharma, 2021; Ruhanya et al., 2022; Wallace, 2022) (Wright et al., 2010; Shikuma et al., 2012). In the present study, we have included the opportunistic infection and the cumulative DDDs of ART drug information between our CHM and non-CHM users and we found that these two groups exhibited similar characteristics. We also found that patients with neurological diseases among HIV/AIDS and CHM users had decreased risks of all-cause mortality and infections, parasites, and circulatory-related mortality. Furthermore, our sensitivity test showed that CHM users still had a lower risk of all-cause mortality than non-CHM users in patients with the CNS infections. Studies have shown that CHMs and associated natural compounds may be beneficial for neurological impairment in HIV/AIDS through anti-cognitive decline *via* the promotion of blood circulation and attenuation of oxidative stress, as well as anti-neuroinflammation and anti-HIV (Han et al., 2011; Cheng et al., 2015; Lin et al., 2015; Esposito et al., 2016; Zhao et al., 2016; Lee et al., 2017; Adebiyi et al., 2018; Long F. Y. et al., 2018; Zhang et al., 2018; Zeng et al., 2019; Qu et al., 2021; Dong et al., 2022; Xu et al., 2022; Xue et al., 2022).

Among herbal formulas, Long-Dan-Xie-Gan-Tang (LDXGT) was the most commonly used herbal formula (prescription frequency: 459). The second, third, and fourth formulas were Ban-Xia-Xie-Xin-Tang (BXXXT) (prescription frequency: 407), Ge-Gen-Tang (GGT) (prescription frequency: 390), and Suan-Zao-Ren-Tang (SZRT) (prescription frequency: 280), respectively. LDXGT is a traditional Chinese herbal formula that is recorded in the ancient Chinese medical text called Yi-Fang-Ji-Jie (Collection of Prescriptions with Notes). It contains 10 Chinese herbs and has a wide range of uses, including the treatment of various types of infectious and inflammatory disorders (Xiong et al., 2018; Fan et al., 2020). In addition, it is also effective and safe against insomnia (Fan et al., 2020). Long Dan Cao (*Gentiana scabra* Bunge) is one herb of LDXGT that shows anti-inflammatory activity, relieves pain, and decreases postherpetic neuralgia in herpes zoster (Wang et al., 2017). Gentianine is a natural compound of Long Dan Cao (root and rhizome of *G. scabra* Bunge) that exhibits anti-ischemic stroke and anti-inflammatory activities (Wang et al., 2021). Baicalein, a natural flavone found in the root of Huang Qin (root of *S. baicalensis* Georgi, a Chinese herb of LDXGT), has antioxidant, anti-neuroinflammatory, and anti-cognitive effects (Jin et al., 2019; Shi et al., 2021). Zhi Zi (*G. jasminoides* J.Ellis) is also one of the aforementioned 10 Chinese herbs of LDXGT that exhibits anti-neuroinflammatory and anti-cognitive impairment effects in cerebral ischemia/reperfusion and Alzheimer’s disease animal models (Liu et al., 2021; Zang et al., 2021; Zang et al., 2022). Crocetin is a natural compound of Zhi Zi (ripe fruit of *G. jasminoides* J.Ellis), which protects neurons against microglial activation (Zang et al., 2022). Vanillic acid is a natural flavone found in the roots of Dang Gui (*Angelica sinensis (Oliv.).* Diels, a Chinese herb of LDXGT, have antioxidant, anti-neuroinflammatory, and anti-cognitive effects (Singh et al., 2015). The Gan Cao (root of *G. uralensis* Fisch.) is another one of the 10 Chinese herbs of LDXGT that exhibits antioxidant and anti-cognitive impairment activities (Ahn et al., 2006).

BXXXT is also a traditional Chinese herbal formula that is recorded in the ancient Chinese medical text called Shang-Han-Lun (The Treatise on Febrile Diseases). It contains seven Chinese herbs and has been used to treat various disorders including gastrointestinal inflammation, metabolic diseases, and depression (Yu et al., 2020; Xia et al., 2022). Zhi Ban Xia (*Pinellia ternata (Thunb.)* Makino) is one herb of BXXXT that promotes sleep by increasing rapid eye movement (REM) sleep (Lin et al., 2019). Gan Jiang (*Zingiber officinale* Roscoe) is one herb of BXXXT that improves cognitive function (Saenghong et al., 2012; Lim et al., 2014). The Gan Cao (root of *G. uralensis* Fisch.) and Huang Qin (root of *S. baicalensis* Georgi), previously reported in LDXGT, were also present in BXXXT. Berberine, one of the main bioactive components of Huang Lian (root of *C. chinensis* Franch., a Chinese herb of BXXXT), has anti-diabetes-related cognitive impairment and anti-cognitive deficiency effects (Durairajan et al., 2012; Hao et al., 2022).

GGT (also called Ge-Gen decoction) is a traditional Chinese herbal formula that is recorded in the ancient Chinese medical text called Shang-Han-Lun (The Treatise on Febrile Diseases) (Chen et al., 2009). It contains seven Chinese herbs and exhibits anti-depression and anti-inflammatory activities (Qin et al., 2019; Chiao et al., 2020). Puerarin, a natural compound of Ge Gen (*Radix Puerariae*, a Chinese herb of GGT), has antioxidant, anti-anxiety, and anti-cognitive effects (Huang et al., 2019; Chiao et al., 2020; Zhu et al., 2021). Ephedrine, a natural compound of Ma Huang (*Ephedrae herba*; a Chinese herb of GGT), has anti-HIV latency activity (Murakami et al., 2008; Panaampon et al., 2019). The Gan Cao (root of *G. uralensis* Fisch.) and Sheng Jiang (*Z. officinale* Roscoe), previously reported in BXXXT, were also present in GGT.

SZRT and YJT (*P. multiflorum* Thunb.) are important CHMs with the strongest connection strength found in this study. SZRT (also called Sansoninto) is a traditional Chinese herbal formula that is recorded in the ancient Chinese medical text called Jin-Gui-Yao-Lue (synopsis of prescriptions of the golden chamber). It contains five Chinese herbs and has been widely used therapeutically for major depressive, anxiety, and sleep disorders (Chen et al., 2011; Yeh et al., 2011; Lee et al., 2013; Xie et al., 2013; Chen et al., 2015; Hu et al., 2015; Ni et al., 2015; Ni et al., 2019; Chen et al., 2021). Jujuboside A and jujuboside B, natural compounds of Suan Zao Ren (*Ziziphus jujuba Mill.*; a Chinese herb of SZRT), have neuroprotective, blood circulation-promoting, and anti-cognitive effects (Seo et al., 2013; Zare-Zardini et al., 2013; Liu et al., 2014; Zhao et al., 2016; Shergis et al., 2017; Zhang et al., 2018; Huang et al., 2019; Zhu et al., 2021). Ye Jiao Teng (YJT) and its related natural compounds (2, 3, 5, and 4′-tetrahydoxystilbene-2-O-β-D-glucoside, emodin, and beta-sitosterol) show antioxidant and anti-cognitive activities (Chan et al., 2003; Um et al., 2006; Lee et al., 2017; Adebiyi et al., 2018; Zeng et al., 2019).

The first CHM cluster included five Chinese herbs: Huang Lian (root of *C. chinensis* Franch), Huang Qin (root of *S. baicalensis* Georgi), Jie Geng (root of *P. grandiflorus* (Jacq.) A.DC.), and Gan Cao (root of *G. uralensis* Fisch.), Huang Bai (bark of *P. amurense* Rupr.). Huang Qin (root of *S. baicalensis* Georgi), Gan Cao (root of *G. uralensis* Fisch.). Huang Lian (root of *C. chinensis* Franch) has also been previously reported in LDXGT, BXXXT, and/or GGT. Jie Geng (root of *P. grandiflorus* (Jacq.) A.DC.) contains platycodin D, which promotes cognitive functions (Kim et al., 2017). Huang Bai (bark of *P. amurense* Rupr.) contains cortex Phellodendri amurensis and exhibits anti-inflammatory activity (Park et al., 2007; Choi et al., 2014).

The limitations of this study are the lack of information on lifestyle, occupation, education, and laboratory tests in the database. However, we observed that CHM may lower the risks of all-cause mortality and infections, parasites, and circulatory-related mortality in patients with neurological diseases, and may be beneficial for functional studies and randomized controlled trials (RCTs) in neurocognitive protection in the future. These CHMs require large-scale RCTs in HIV/AIDS patients with neurological diseases to confirm their safety and relative effectiveness and to plot their interactions during regular treatments in these patients.

Among HIV/AIDS patients with neurological diseases, CHM users showed a better survival rate. Based on network analysis and association rule mining, the two CHM clusters were identified as potential CHMs for these patients. Further studies are required to validate the efficacy and safety of CHMs in these patients. The mechanism of the interactions between the natural compounds of CHMs also requires further investigation.

## Data Availability

The data analyzed in this study is subject to the following licenses/restrictions: Only citizens of the Republic of China who fulfill the requirements of conducting research projects are eligible to apply for the National Health Insurance Research Database (NHIRD). The use of NHIRD is limited to research purposes only. Applicants must follow the Computer-Processed Personal Data Protection Law (http://www.winklerpartners.com/?p&equals;987) and related regulations of National Health Insurance Administration and NHRI (National Health Research Institutes), and an agreement must be signed by the applicant and his/her supervisor upon application submission. All applications are reviewed for approval of data release. Requests to access these datasets should be directed to Y-JL, yjlin.kath@gmail.com.
