# Supplementary material for: Effect of Chinese herbal medicine therapy on risks of all-cause mortality, infections, parasites, and circulatory-related mortality in HIV/AIDS patients with neurological diseases
Source: Front Pharmacol. 2023 Mar 3;14:1097862. doi: 10.3389/fphar.2023.1097862 (PMC10020503; doi:10.3389/fphar.2023.1097862)
Supplement: Supplementary file 1 [file DataSheet1.docx]

**Supplementary Information**

Supplementary Text

Figures S1-S6

Tables S1-S10

**Supplementary Text**

**Figure S1** | Cumulative incidence of all-cause mortality between CHM and non-CHM users in HIV/AIDS patients with neurological diseases (Cumulative days of CHM treatment ≧28 days within the first year after neurological diseases diagnosis) in Taiwan. Abbreviations: CHM, Chinese herbal medicine.

**Figure S2** | Cumulative incidence of all-cause mortality between CHM and non-CHM users in HIV/AIDS patients with neurological diseases (Cumulative days of CHM treatment ≧84 days within the first year after neurological diseases diagnosis) in Taiwan. Abbreviations: CHM, Chinese herbal medicine.

**Figure S3** | Frequency distribution of neurological disease subtypes among patients with HIV/AIDS.

**Figure S4** | Cumulative incidence of all-cause mortality between CHM and non-CHM users in HIV/AIDS patients with the CNS infections. Abbreviations: CHM, Chinese herbal medicine.

**Figure S5** | CHM cluster 1 and its prescription compatibility for neurological diseases including single herb, modern medicine symptom, neurological diseases using SymMap website (http://www.symmap.org/). Abbreviations: HB, Huang Bai (bark of *Phellodendron amurense* Rupr.); JG, Jie Geng (root of *Platycodon grandiflorus* (Jacq.) A.DC.); GC, Gan Cao (root of *Glycyrrhiza uralensis* Fisch.); HL, Huang Lian (root of *Coptis chinensis* Franch.); HQ, Huang Qin (root of *Scutellaria baicalensis* Georgi).

**Figure S6** | CHM cluster 2 and its prescription compatibility for neurological diseases including single herb, modern medicine symptom, neurological diseases using SymMap website (http://www.symmap.org/). Abbreviations: SZRT, Suan-Zao-Ren-Tang; ZM, Zhi Mu (rhizomes of *Anemarrhena asphodeloides* Bunge.); SZR, Suan Zao Ren (seed of *Ziziphus jujuba* Mill.); FL, Fu Ling (sclerotium of *Wolfiporia extensa* (Peck) Ginns); ChuanX, Chuan Xiong (rhizome of *Ligusticum sinense* Oliv.); GC, Gan Cao (root of *Glycyrrhiza uralensis* Fisch.); YJT, Ye Jiao Teng (stem of *Polygonum multiflorum* Thunb.).

**TABLE S1 |** Composition of the most commonly used herbal formulas and single herbs for patients with neurological diseases among HIV/AIDS in Taiwan.

**TABLE S2 |** Therapeutic actions and indications of the most commonly used herbal formulas and single herbs for patients with neurological diseases among HIV/AIDS in Taiwan.

**TABLE S3** | Cox proportional hazard models for risk of overall mortality in patients with neurological diseases among HIV/AIDS.

**TABLE S4** | Distribution of the cumulative CHM treatment days during the study period among HIV/AIDS patients with neurological diseases.

**TABLE S5** | Cox proportional hazard models for risk of overall mortality in patients with neurological diseases among HIV/AIDS (cumulative CHM treatment days ≧28 days within the first year after neurological diseases).

**TABLE S6** | Cox proportional hazard models for risk of overall mortality in patients with neurological diseases among HIV/AIDS (cumulative CHM treatment days ≧84 days within the first year after neurological diseases).

**TABLE S7** | Number and percent distribution of inpatient and outpatient visits within one year after the diagnosis of HIV/AIDS.

**TABLE S8** | Malignancy distribution of patients with neurological diseases among HIV/AIDS according to Chinese herbal medicine usage in Taiwan.

**TABLE S9** | Cause of death of patients with neurological diseases among HIV/AIDS according to ICD-9-CM and ICD-10-CM codes in Taiwan.

**TABLE S10** | Cox proportional hazard models for risk of overall mortality in patients with the CNS infections among HIV/AIDS.

**Figure S1**


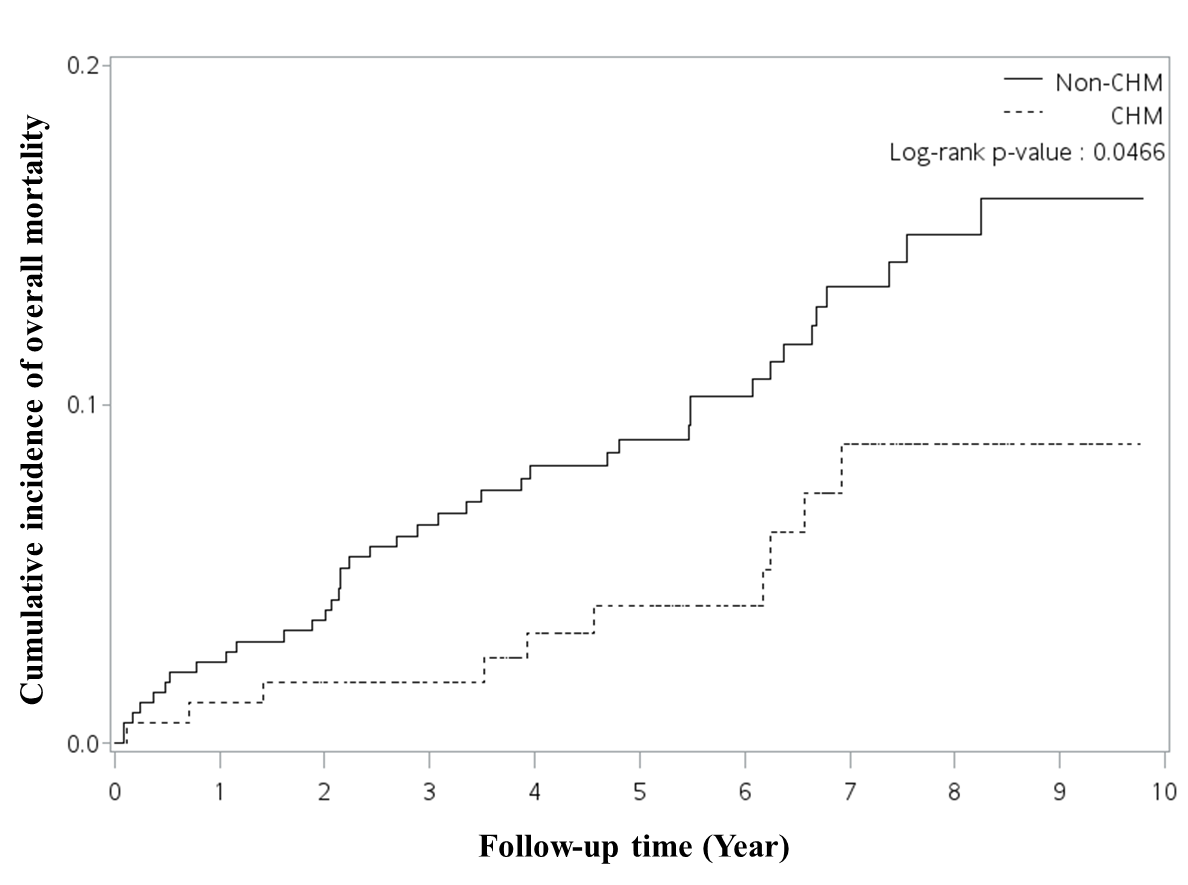


**Figure S2**


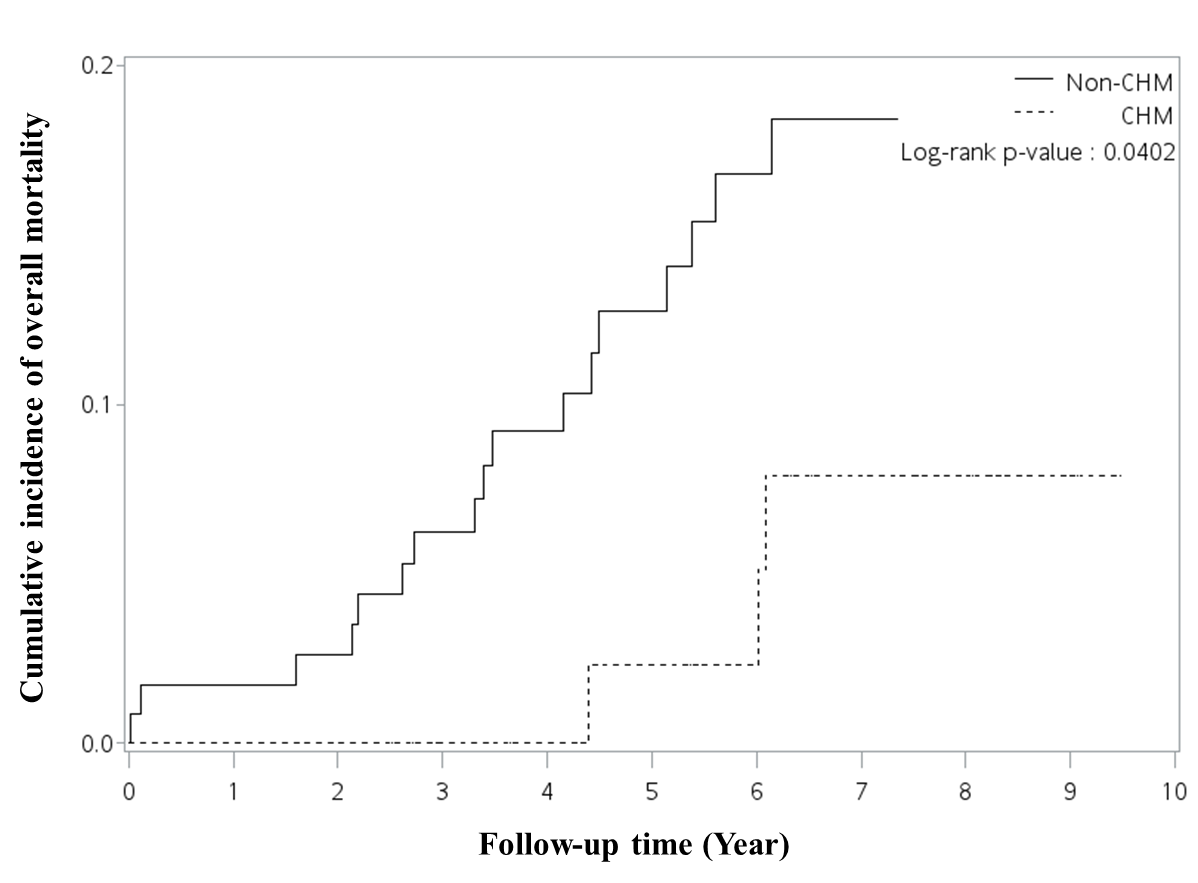


**Figure S3**


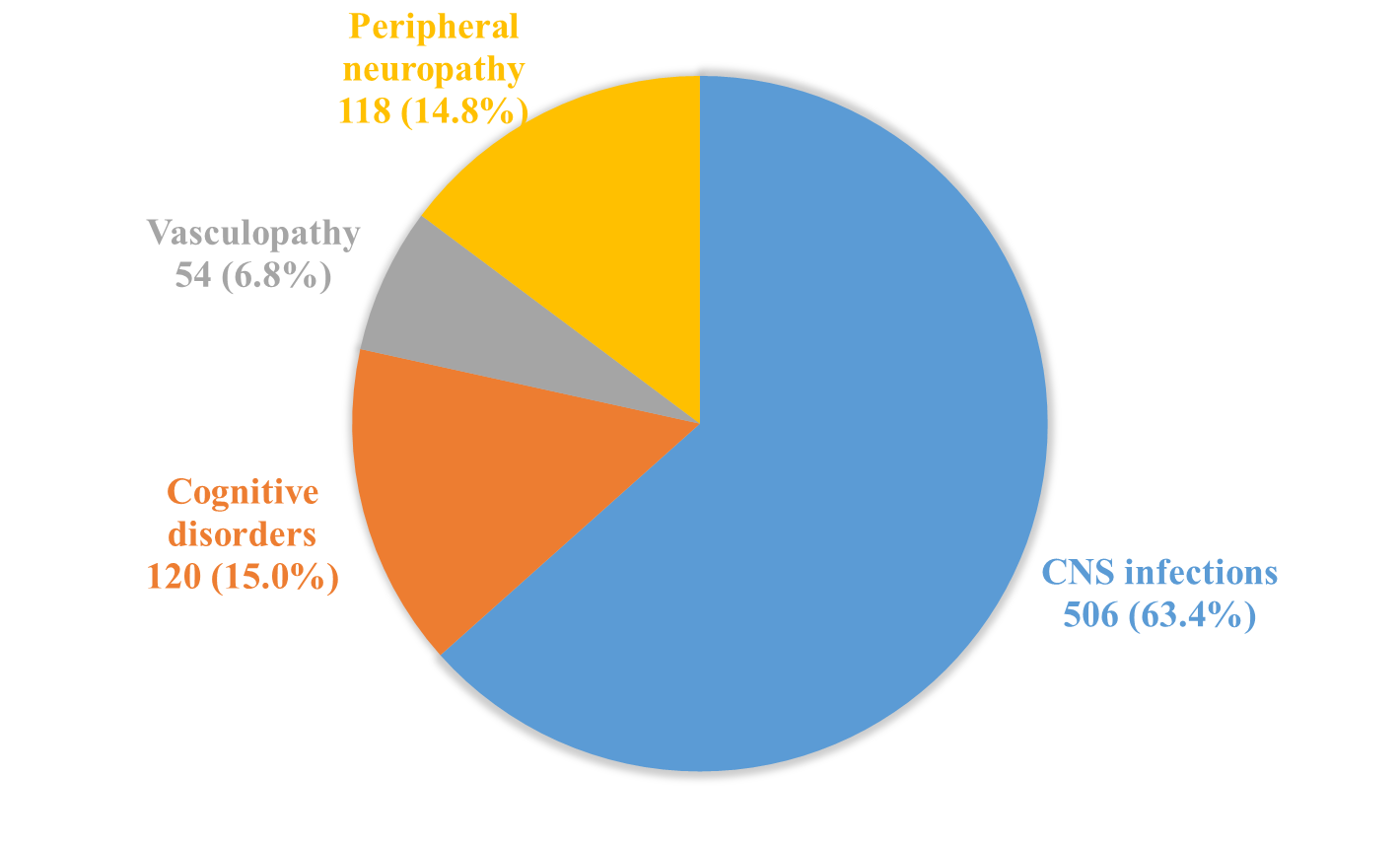


**Figure S4**


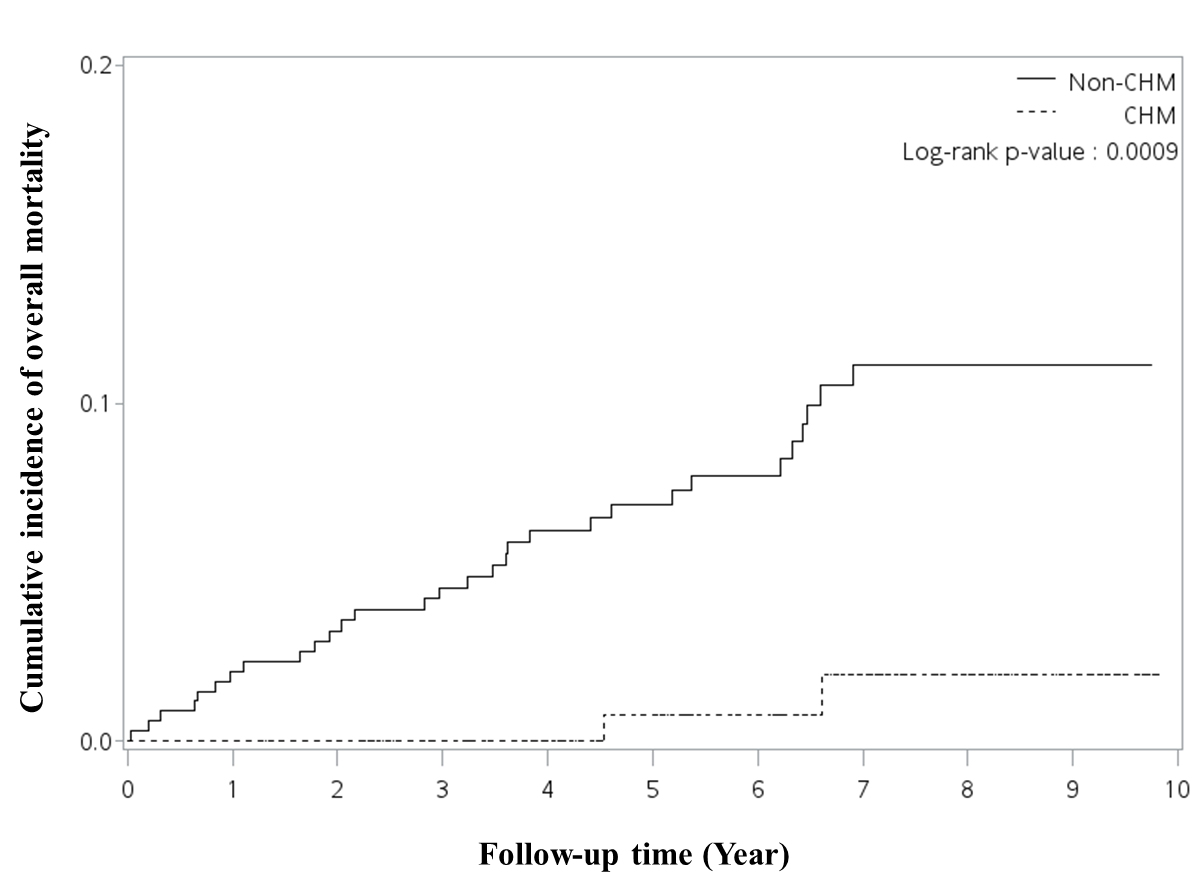


**Figure S5**


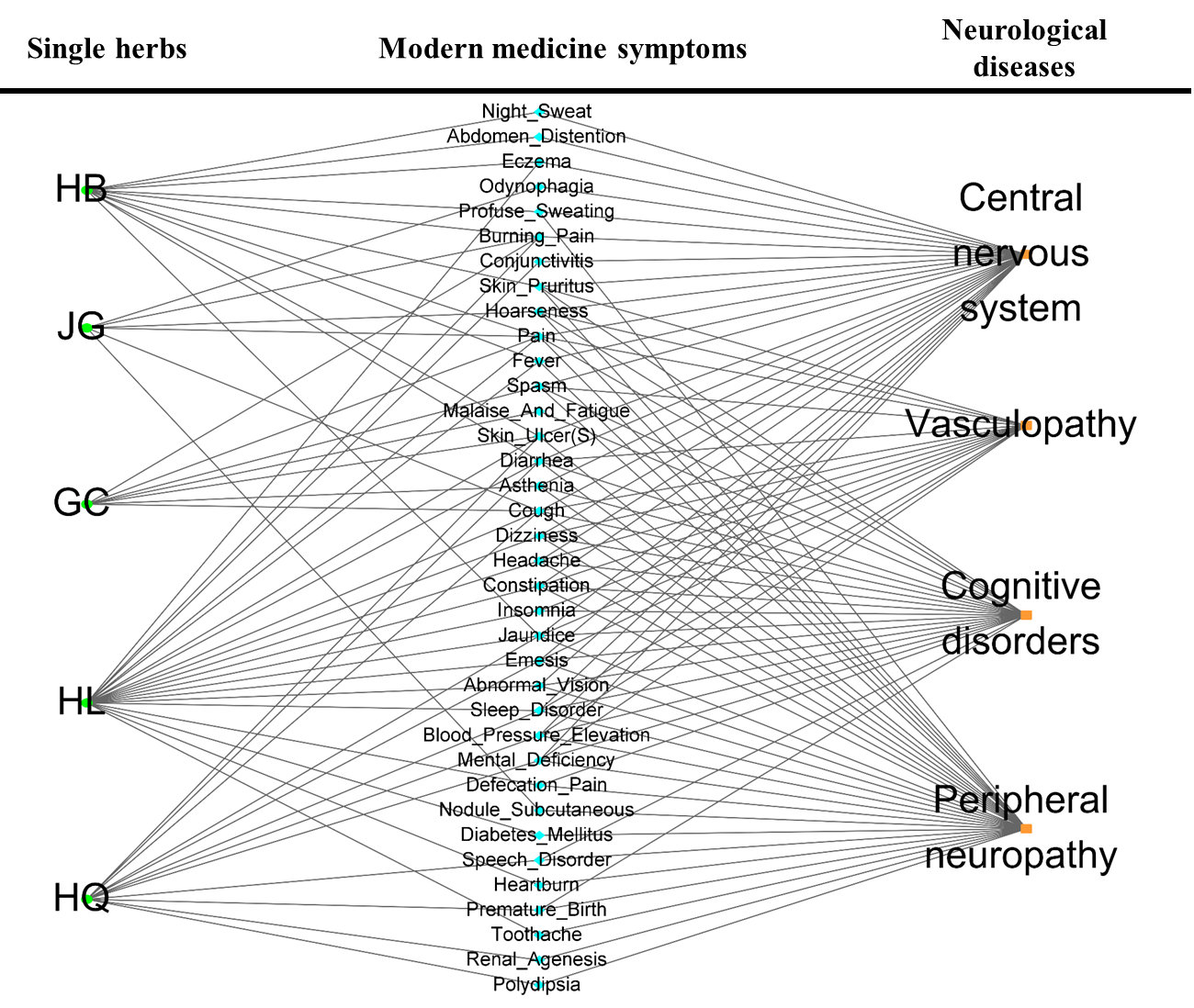


**Figure S6**


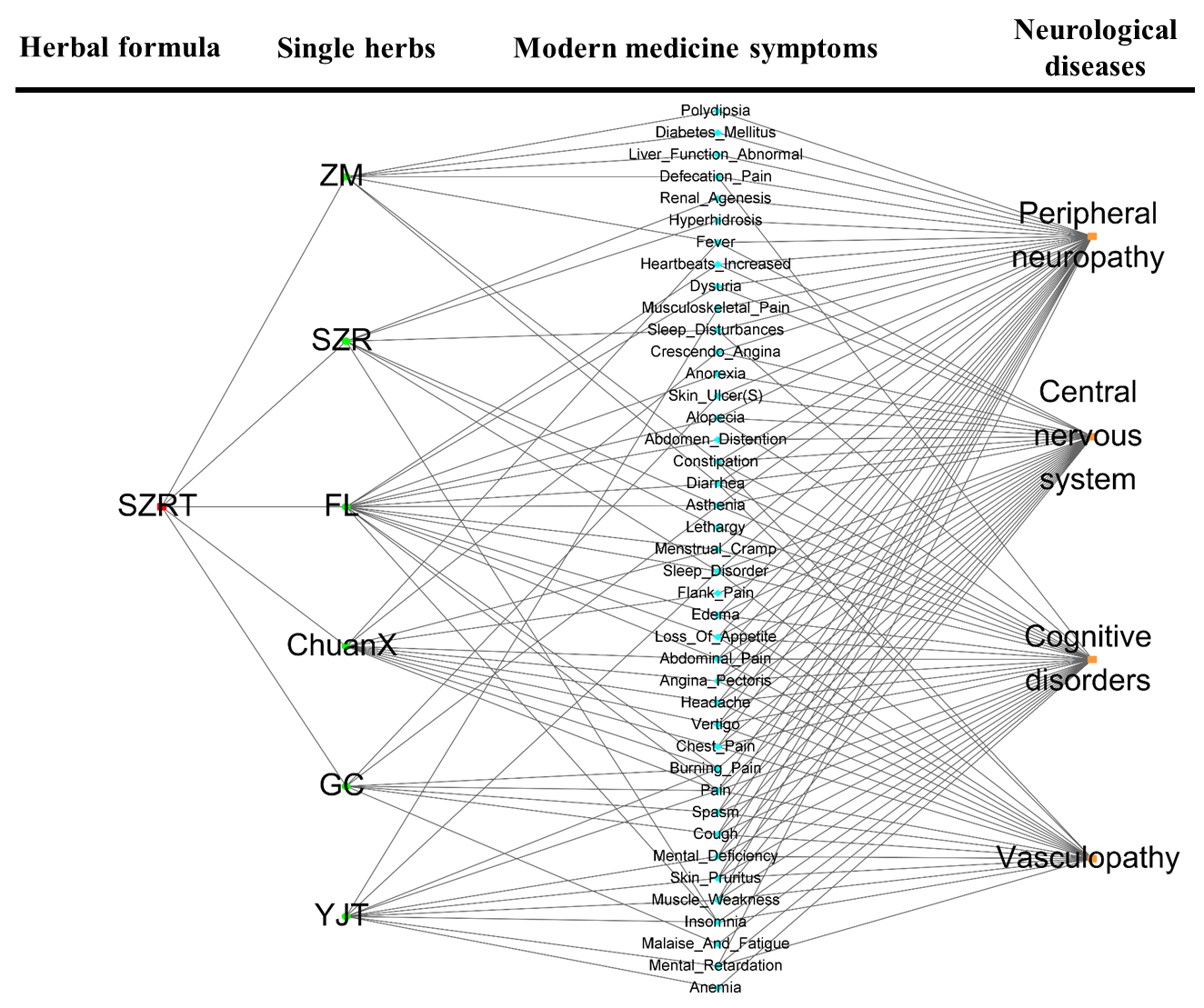


| **TABLE S1 \| Composition of the most commonly used herbal formulas and single herbs for patients with neurological diseases among HIV/AIDS in Taiwan** | | | | | | | |
| --- | --- | --- | --- | --- | --- | --- | --- |
| **Formulas** | **Chinese name** | **Total number of herbs** | **Frequency of prescriptions** | **Person-year** | **Percentage of usage person** | **Avg. drug dose per day (g)** | **Average duration for prescription (days)** |
| **Total** |  |  | **7376** | **1618.50** | **100.00** | **12.91** | **7.62** |
| **Herbal formula (Pin-yin name)** |  |  | **7022** | **1612.30** | **99.62** | **9.60** | **7.55** |
| Long-Dan-Xie-Gan-Tang (LDXGT) | 龍膽瀉肝湯 | 10 | 459 | 576.90 | 32.33 | 3.79 | 7.56 |
| Ban-Xia-Xie-Xin-Tang (BXXXT) | 半夏瀉心湯 | 7 | 407 | 522.90 | 30.45 | 3.88 | 7.15 |
| Ge-Gen-Tang (GGT) | 葛根湯 | 7 | 390 | 594.70 | 34.21 | 4.89 | 6.91 |
| Suan-Zao-Ren-Tang (SZRT) | 酸棗仁湯 | 5 | 280 | 406.90 | 23.31 | 4.10 | 7.17 |
|  |  |  |  |  |  |  |  |
| **Single herbs (Pin-yin name)** |  |  | **6102** | **1571.70** | **96.99** | **4.70** | **7.55** |
| Huang Qin (HQ) | 黃芩 | 1 | 557 | 625.60 | 34.96 | 1.30 | 8.26 |
| Gan Cao (GC) | 甘草 | 1 | 540 | 578.50 | 33.46 | 1.02 | 7.53 |
| Da Huang (DaH) | 大黃 | 1 | 528 | 409.10 | 22.93 | 0.40 | 7.78 |
| Jie Geng (JG) | 桔梗 | 1 | 466 | 592.00 | 33.83 | 1.14 | 6.87 |
| Huang Lian (HL) | 黃連 | 1 | 399 | 403.00 | 21.80 | 0.97 | 6.95 |
| Ye Jiao Teng (YJT) | 夜交藤 | 1 | 324 | 504.00 | 28.95 | 1.21 | 7.08 |
| Huang Bai (HB) | 黃柏 | 1 | 280 | 461.70 | 24.44 | 1.27 | 7.14 |
| *Sorted by frequency of prescriptions. | | | | | | | |
| Information are obtained from the websites (http://www.americandragon.com/index.htm; http://old.tcmwiki.com/; http://www.shen-nong.com/eng/front/index.html; http://www.ipni.org/; http://www.theplantlist.org/). | | | | | | | |

| **TABLE S2 \| Therapeutic actions and indications of the most commonly used herbal formulas and single herbs for patients with neurological diseases among HIV/AIDS in Taiwan** | | | | | | | | |
| --- | --- | --- | --- | --- | --- | --- | --- | --- |
| **Formulas** | **Chinese name** | **Total number of herbs** | **Number** | **Composition** | | | | **Therapeutic actions and indications** |
|  |  |  |  | **Pin-yin name from American Dragon** | **Chinese name for single herb** | **Latin name** | **Botanical plant name** |  |
|  |  |  |  | **(http://www.americandragon.com/index.htm)** |  |  |  |  |
| **Total** |  |  |  |  |  |  |  |  |
| **Herbal formula (Pin-yin name)** |  |  |  |  |  |  |  |  |
| Long-Dan-Xie-Gan-Tang (LDXGT) | 龍膽瀉肝湯 | 10 | 1 | Long Dan Cao | 龍膽草 | *Radix Gentianae* | *Gentiana scabra* Bunge | Anti-oxidant, anti-neuroinflammatory, anti-insomnia, anti-ischemic stroke, anti-cognitive deficiency activities (Fan et al., 2020; Wang et al., 2017; Wang et al., 2021; Shi et al., 2021; Jin et al., 2019; Liu et al., 2021;Zang et al., 2021;Zang et al., 2022; Singh et al., 2015; Ahn et al., 2006) |
|  |  |  | 2 | Huang Qin | 黄芩 | *Radix Scutellariae* | *Scutellaria baicalensis* Georgi |  |
|  |  |  | 3 | Zhi Zi | 梔子 | *Fructus Gardeniae* | *Gardenia jasminoides* J.Ellis |  |
|  |  |  | 4 | Mu Tong | 木通 | *Caulis Akebiae* | *Akebia chingshuiensis* T.Shimizu |  |
|  |  |  | 5 | Che Qian Zi | 車前子 | *Semen Plantaginis* | *Plantago depressa* Willd. |  |
|  |  |  | 6 | Ze Xie | 澤瀉 | *Rhizoma Alismatis* | *Alisma* plantago-aquatica L. |  |
|  |  |  | 7 | Chai Hu | 柴胡 | *Radix Bupleuri* | *Bupleurum* chinense DC. |  |
|  |  |  | 8 | Sheng Di Huang | 地黃 | *Radix Rehmanniae* | *Rehmannia glutinosa* (Gaertn.) DC. |  |
|  |  |  | 9 | Dang Gui | 當歸 | *Radix Angelicae Sinensi* | *Angelica sinensis* (Oliv.) Diels |  |
|  |  |  | 10 | Gan Cao | 甘草 | *Radix Glycyrrhizae* | *Glycyrrhiza uralensis* Fisch. |  |
|  |  |  |  |  |  |  |  |  |
| Ban-Xia-Xie-Xin-Tang (BXXXT) | 半夏瀉心湯 | 7 | 1 | Zhi Ban Xia | 半夏 | *Rhizoma Pinelliae Preparatum* | *Pinellia ternata (Thunb.)* Makino | Promoting sleep, improving cognitive function, anti-oxidant, anti-neuroinflammatory, and anti-cognitive deficiency, anti-diabetes related cognitive impairement (Lin et al., 2019; Saenghong et al., 2012;Lim et al., 2014; Jin et al., 2019;Shi et al., 2021; Ahn et al., 2006; Durairajan et al., 2012;Hao et al., 2022) |
|  |  |  | 2 | Gan Jiang | 干姜 | *Rhizoma Zingiberis* | *Zingiber officinale* Roscoe |  |
|  |  |  | 3 | Huang Qin | 黄芩 | *Radix Scutellariae* | *Scutellaria baicalensis* Georgi |  |
|  |  |  | 4 | Huang Lian | 黃連 | *Rhizoma Coptidis* | *Coptis chinensis* Franch. |  |
|  |  |  | 5 | Ren Shen | 人參 | *Radix Ginseng* | *Panax ginseng* C.A.Mey. |  |
|  |  |  | 6 | Da Zao | 大棗 | *Fructus Jujube* | *Ziziphus jujuba* Mill. |  |
|  |  |  | 7 | Gan Cao | 甘草 | *Radix Glycyrrhizae* | *Glycyrrhiza uralensis* Fisch. |  |
|  |  |  |  |  |  |  |  |  |
| Ge-Gen-Tang (GGT) | 葛根湯 | 7 | 1 | Ge Gen | 葛根 | *Radix Puerariae* | *Pueraria lobata (Willd.)* Ohwi | Anti-inflammatory, anti-oxidant, anti-anxiety, anti-cognitive impairment, anti-HIV latency, and improving cognitive function effects (Qin et al., 2019; (Huang et al., 2019; Zhu et al., 2021; Murakami et al., 2008;Panaampon et al., 2019; Saenghong et al., 2012;Lim et al., 2014; Ahn et al., 2006) |
|  |  |  | 2 | Ma Huang | 麻黃 | *Herba Ephedrae* | *Ephedra vulgaris* Rich. |  |
|  |  |  | 3 | Gui Zhi | 桂枝 | *Cinnamomi ramulus* | *Cinnamomum cassia (L.)* J.Presl |  |
|  |  |  | 4 | Bai Shao | 白芍 | *Radix Paeoniae Alba* | *Paeonia lactiflora* Pall. |  |
|  |  |  | 5 | Sheng Jiang | 生姜 | *Rhizoma Zingiberis Recens* | *Zingiber officinale* Roscoe |  |
|  |  |  | 6 | Da Zao | 大棗 | *Fructus Jujube* | *Ziziphus jujuba* Mill. |  |
|  |  |  | 7 | Gan Cao | 甘草 | *Radix Glycyrrhizae* | *Glycyrrhiza uralensis* Fisch. |  |
|  |  |  |  |  |  |  |  |  |
| Suan-Zao-Ren-Tang (SZRT) | 酸棗仁湯 | 5 | 1 | Suan Zao Ren | 酸棗仁 | *Semen Zizyphi Spinosae* | *Ziziphus jujuba* Mill. | Anti-anxiety, anti-depression, anti-cognitive deficiency, and promoting blood circulation activities (Liu et al., 2014; Zhang, M. et al., 2018; Seo et al., 2013; Zhao et al., 2016; Zare-Zardini et al., 2013; Liu et al., 2012; Kuang et al., 2017; Li et al., 2015; Long et al., 2018; Xu et al., 2018) |
|  |  |  | 2 | Fu Ling | 茯苓 | *Poria* | *Wolfiporia extensa* (Peck) Ginns |  |
|  |  |  | 3 | Zhi Mu | 知母 | *Rhizoma Anemarrhenae* | *Anemarrhena asphodeloides* Bunge. |  |
|  |  |  | 4 | Chuan Xiong | 川芎 | *Rhizoma Ligustici* | *Ligusticum sinense* Oliv. |  |
|  |  |  | 5 | Gan Cao | 甘草 | *Radix Glycyrrhizae* | *Glycyrrhiza uralensis* Fisch. |  |
|  |  |  |  |  |  |  |  |  |
| **Single herbs (Pin-yin name)** |  |  |  |  |  |  |  |  |
| Huang Qin (HQ) | 黃芩 | 1 | 1 | Huang Qin | 黃芩 | *Radix Scutellariae* | *Scutellaria baicalensis* Georgi | Anti-oxidant, anti-neuroinflammatory, and anti-cognitive activities (Jin et al., 2019;Shi et al., 2021) |
| Gan Cao (GC) | 甘草 | 1 | 1 | Gan Cao | 甘草 | *Radix Glycyrrhizae* | *Glycyrrhiza uralensis* Fisch. | Anti-oxidant and anti-cognitive impairment activities (Ahn et al., 2006) |
| Jie Geng (JG) | 桔梗 | 1 | 1 | Jie Geng | 桔梗 | *Radix Platycodi* | *Platycodon grandiflorus* (Jacq.) A.DC. | Promoting cognitive function (Kim et al., 2017) |
| Huang Lian (HL) | 黃連 | 1 | 1 | Huang Lian | 黃連 | *Rhizoma Coptidis* | *Coptis chinensis* Franch. | Anti-diabetes related cognitive impairement and anti-cognitive deficiency effects (Durairajan et al., 2012;Hao et al., 2022) |
| Ye Jiao Teng (YJT) | 夜交藤 | 1 | 1 | Ye Jiao Teng | 夜交藤 | *Caulis Polygoni Multiflori* | *Polygonum multiflorum* Thunb. | Anti-cognitive deficiency and anti-oxidant activities (Lee et al., 2017; Zeng et al., 2019; Adebiyi et al., 2018) |
| Huang Bai (HB) | 黃柏 | 1 | 1 | Huang Bai | 黃柏 | *Cortex Phellodendri* | *Phellodendron amurense* Rupr. | Anti-inflammatory activity (Park et al., 2007;Choi et al., 2014) |
| *Sorted by frequency of prescriptions. NA, not available. | | | | | | | | |
| Information are obtained from the websites (http://www.americandragon.com/index.htm; http://old.tcmwiki.com/; http://www.shen-nong.com/eng/front/index.html; http://www.ipni.org/; http://www.theplantlist.org/). | | | | | | | | |

| **TABLE S3 \| Cox proportional hazard models for risk of overall mortality in patients with neurological diseases among HIV/AIDS** | | | | | | | |
| --- | --- | --- | --- | --- | --- | --- | --- |
|  | **Overall mortality** | | | | | | |
|  | **Crude** | | |  | **Adjusted** | | |
|  | **HR** | **95% CI** | ***p*-value** |  | **aHR** | **95% CI** | ***p*-value** |
| **Age (years old)** |  |  |  |  |  |  |  |
| 30≦Age<40 (vs. 0≦Age<30) | 1.49 | (0.83-2.68) | 0.185 |  | 1.22 | (0.66-2.25) | 0.529 |
| 40≦Age (vs. 0≦Age<30) | 4.48 | (2.64-7.60) | ***<0.001*** |  | 3.73 | (2.11-6.60) | ***<0.001*** |
| **Female (vs. male)** | 2.98 | (1.40-6.32) | ***0.005*** |  | 3.02 | (1.34-6.79) | ***0.008*** |
| **CHM use (vs. non-CHM use)** | 0.38 | (0.21-0.66) | ***<0.001*** |  | 0.31 | (0.16-0.59) | ***<0.001*** |
| **Charlson comorbidity number_1-2 (vs. 0)** | 1.80 | (1.05-3.10) | ***0.033*** |  | 1.36 | (0.75-2.49) | 0.311 |
| **Charlson comorbidity number_≥3 (vs. 0)** | 3.90 | (2.00-7.64) | ***<0.001*** |  | 1.97 | (0.94-4.13) | 0.071 |
| HR, hazard ratio; aHR, adjusted hazard ratio; CI, confidence interval; CHM, Chinese herbal medicine. | | | | | | | |
| Adjusted factors: age, sex, CHM use, Charlson comorbidity number, and interval (between the date of neurological diseases and index date). | | | | | | | |
| The date after the 14 days’ cumulative prescription of CHM was designated the index date. | | | | | | | |
| Significant *p*-values (*p* < 0.05) are highlighted in bold italic font. | | | | | | | |

| **TABLE S4 \|** Distribution of the cumulative CHM treatment days during the study period among HIV/AIDS patients with neurological diseases | | | |
| --- | --- | --- | --- |
| **Cumulative CHM treatment days within the first year after neurological diseases** | **Cumulative CHM treatment days during the study period (started after the index date)** | **CHM users** | |
|  |  | **N** | **%** |
| **≥14 days (N = 266)** |  |  | |
|  | **day < 180** | 179 | 67.3 |
|  | **180 ≤ day< 360** | 42 | 15.8 |
|  | **day ≥ 360** | 45 | 16.9 |
| **≥28 days (N = 170)** |  |  |  |
|  | **day < 180** | 92 | 54.1 |
|  | **180 ≤ day< 360** | 37 | 21.8 |
|  | **day ≥ 360** | 41 | 24.1 |
| **≥84 days (N = 60)** |  |  |  |
|  | **day < 180** | 16 | 26.7 |
|  | **180 ≤ day< 360** | 17 | 28.3 |
|  | **day ≥ 360** | 27 | 45.0 |
| N, number; CHM, Chinese herbal medicine. | | | |
| The index date of this study was from the day on which the 14, 28, or 84 cumulative days of CHM treatment with the first year were completed. | | | |
| Cumulative CHM treatment days during the study period was started after the index date. | | | |

| **TABLE S5 \| Cox proportional hazard models for risk of overall mortality in patients with neurological diseases among HIV/AIDS (cumulative CHM treatment days ≧28 days within the first year after neurological diseases)** | | | | | | | |
| --- | --- | --- | --- | --- | --- | --- | --- |
|  | **Overall mortality** | | | | | | |
|  | **Crude** | | |  | **Adjusted** | | |
|  | **HR** | **95% CI** | ***p*-value** |  | **aHR** | **95% CI** | ***p*-value** |
| **Age (years old)** |  |  |  |  |  |  |  |
| 30≦Age<40 (vs. 0≦Age<30) | 1.99 | (0.96-4.13) | 0.065 |  | 1.84 | (0.87-3.87) | 0.108 |
| 40≦Age (vs. 0≦Age<30) | 3.54 | (1.79-6.98) | ***<0.001*** |  | 3.13 | (1.55-6.32) | ***0.002*** |
| **Female (vs. male)** | 1.20 | (0.36-4.03) | 0.766 |  | 0.81 | (0.22-3.03) | 0.757 |
| **CHM use (vs. non-CHM use)** | 0.50 | (0.24-1.04) | 0.063 |  | 0.46 | (0.22-0.96) | ***0.038*** |
| **Charlson comorbidity number_1-2 (vs. 0)** | 1.86 | (0.99-3.47) | 0.053 |  | 1.49 | (0.78-2.85) | 0.227 |
| **Charlson comorbidity number_≥3 (vs. 0)** | 2.81 | (1.12-7.08) | ***0.028*** |  | 2.13 | (0.8-5.68) | 0.129 |
| HR, hazard ratio; aHR, adjusted hazard ratio; CI, confidence interval; CHM, Chinese herbal medicine. | | | | | | | |
| Adjusted factors: age, sex, CHM use, and Charlson comorbidity number. | | | | | | | |
| The date after the 28 days cumulative prescription of CHM was designated the index date. | | | | | | | |
| Significant *p*-values (*p* < 0.05) are highlighted in bold italic font. | | | | | | | |

| **TABLE S6 \| Cox proportional hazard models for risk of overall mortality in patients with neurological diseases among HIV/AIDS (cumulative CHM treatment days ≧84 days within the first year after neurological diseases)** | | | | | | | |
| --- | --- | --- | --- | --- | --- | --- | --- |
|  | **Overall mortality** | | | | | | |
|  | **Crude** | | |  | **Adjusted** | | |
|  | **HR** | **95% CI** | ***p*-value** |  | **aHR** | **95% CI** | ***p*-value** |
| **Age (years old)** |  |  |  |  |  |  |  |
| 30≦Age<40 (vs. 0≦Age<30) | 2.37 | (0.63-8.88) | 0.201 |  | 2.07 | (0.48-8.86) | 0.327 |
| 40≦Age (vs. 0≦Age<30) | 9.41 | (2.83-31.29) | ***<0.001*** |  | 6.83 | (1.9-24.6) | ***0.003*** |
| **Female (vs. male)** | 2.29 | (0.49-10.77) | 0.295 |  | 1.85 | (0.34-10.23) | 0.480 |
| **CHM use (vs. non-CHM use)** | 0.30 | (0.09-1.02) | 0.053 |  | 0.26 | (0.06-1.07) | 0.062 |
| **Charlson comorbidity number_1-2 (vs. 0)** | 2.84 | (1.16-6.94) | ***0.022*** |  | 2.37 | (0.94-5.99) | 0.068 |
| **Charlson comorbidity number_≥3 (vs. 0)** | 3.89 | (1.65-9.17) | ***0.002*** |  | 1.44 | (0.44-4.66) | 0.546 |
| HR, hazard ratio; aHR, adjusted hazard ratio; CI, confidence interval; CHM, Chinese herbal medicine. | | | | | | | |
| Adjusted factors: age, sex, CHM use, and Charlson comorbidity number. | | | | | | | |
| The date after the 84 days cumulative prescription of CHM was designated the index date. | | | | | | | |
| Significant *p*-values (*p* < 0.05) are highlighted in bold italic font. | | | | | | | |

| **TABLE S7 \| Number and percent distribution of inpatient and outpatient visits within one year after the diagnosis of HIV/AIDS** | | | |
| --- | --- | --- | --- |
| **No.** | **Type of clinic** | **Number of visits** | **Percentage (number of visits)** |
| **1** | Infectious Disease | 714 | 50.53 |
| **2** | Internal Medicine | 235 | 16.63 |
| **3** | Emergency Medicine | 96 | 6.79 |
| **4** | Psychiatry | 67 | 4.74 |
| **5** | Thoracic Medicine | 36 | 2.55 |
| **6** | Neurology | 34 | 2.41 |
| **7** | Dermatology | 26 | 1.84 |
| **8** | Surgery | 21 | 1.49 |
| **9** | Colorectal Surgery | 21 | 1.49 |
| **10** | Gastroenterology | 20 | 1.42 |
| **11** | Ophthalmology | 16 | 1.13 |
| **12** | Orthopedic | 13 | 0.92 |
| **13** | Otorhinolaryngology | 12 | 0.85 |
| **14** | Rheumatology and Immunology | 12 | 0.85 |
| **15** | Neurosurgery | 10 | 0.71 |
| **16** | Family Medicine | 9 | 0.64 |
| **17** | Urology | 8 | 0.57 |
| **18** | Rehabilitation | 8 | 0.57 |
| **19** | Nephrology | 7 | 0.5 |
| **20** | Gastrointestinal Surgery | 7 | 0.5 |
| **21** | Tuberculosis | 6 | 0.42 |
| **22** | Cardiology and Vascular Medicine | 5 | 0.35 |
| **23** | Thoracic Surgery | 5 | 0.35 |
| **24** | Obstetrics and Gynecology | 3 | 0.21 |
| **25** | Thoracic and Critical Care Medicine | 3 | 0.21 |
| **26** | Home Care | 3 | 0.21 |
| No., number. | | | |
| Total visits = 1413 within one year after the diagnosis of HIV/AIDS. | | | |

| **TABLE S8 \| Malignancy distribution of patients with neurological diseases among HIV/AIDS according to Chinese herbal medicine usage in Taiwan** | | | | | | |
| --- | --- | --- | --- | --- | --- | --- |
| **Malignancy** | **Included subjects** | | ***p*-value** | **Matched subjects** | | ***p*-value** |
|  | **CHM users (N = 308)** | **Non-CHM users (N = 901)** |  | **CHM users (N = 266)** | **Non-CHM users (N = 532)** |  |
|  | **N (%)** | **N (%)** |  | **N (%)** | **N (%)** |  |
| Kaposi’s sarcoma |  |  | ND |  |  | ND |
| No | 308 ( 100%) | 901 ( 100%) |  | 266 ( 100%) | 532 ( 100%) |  |
| Yes | 0 | 0 |  | 0 | 0 |  |
| Lymphoma |  |  | ND |  |  | ND |
| No | 308 ( 100%) | 901 ( 100%) |  | 266 ( 100%) | 532 ( 100%) |  |
| Yes | 0 | 0 |  | 0 | 0 |  |
| Invasive cervical cancer |  |  | ND |  |  | ND |
| No | 308 ( 100%) | 901 ( 100%) |  | 266 ( 100%) | 532 ( 100%) |  |
| Yes | 0 | 0 |  | 0 | 0 |  |
| N, number; Chinese herbal medicine; ND, not determined. | | | | | | |
| Malignancy was followed up during the study period. | | | | | | |
| Kaposi’s sarcoma: the ICD-9-CM code: 176 and the ICD-10-CM code: C46.X; lymphoma: the ICD-9-CM code: 200.2 and the ICD-10-CM code: C83.7X; invasive cervical cancer : the ICD-9-CM code: 180 and the ICD-10-CM code: C53.X. | | | | | | |

| **TABLE S9 \| Cause of death of patients with neurological diseases among HIV/AIDS according to ICD-9-CM and ICD-10-CM codes in Taiwan** | | | | | |
| --- | --- | --- | --- | --- | --- |
| **No.** | **Cause of death** | **ICD-9-CM code** | **ICD-10-CM code** | **Overall (Number = 82)** | |
|  |  |  |  | **Number of death** | **Percentage (number of death)** |
| **1** | Infections and parasites | 001-139 | A00-B99 | 31 | 37.8% |
| **2** | Circulatory | 390-459 | I00-I99 | 11 | 13.4% |
| **3** | Endocrine, nutritional, & metabolic diseases | 240-279 | E00-E90 | 4 | 4.9% |
| **4** | Viral hepatitis, and liver diseases | 070, 570-573 | B15-B19, K70-K77 | 4 | 4.9% |
| **5** | Respiratory | 460-519 | J00-J99 | 4 | 4.9% |
| **6** | Genitourinary system | 580-629 | N00-N99 | 0 | 0.0% |
| **7** | Neoplasms | 140-239 | C00-D48 | 0 | 0.0% |
| **8** | Other causes | All codes not previously listed | All codes not previously listed | 28 | 34.1% |
|  | Total |  |  | 82 | 100.0% |
| No., number; ICD-9-CM, International Classification of Disease, 9th Revision, Clinical Modification; ICD-10-CM, International Classification of Disease, 10th Revision, Clinical Modification. | | | | | |
| Total deaths = 82 for patients with neurological diseases among HIV/AIDS. | | | | | |
| References: PMID: 30421717 and PMID: 28241797. | | | | | |

| **TABLE S10 \| Cox proportional hazard models for risk of overall mortality in patients with the CNS infections among HIV/AIDS** | | | | | | | |
| --- | --- | --- | --- | --- | --- | --- | --- |
|  | **Overall mortality** | | | | | | |
|  | **Crude** | | |  | **Adjusted** | | |
|  | **HR** | **95% CI** | ***p*-value** |  | **aHR** | **95% CI** | ***p*-value** |
| **Age (years old)** |  |  |  |  |  |  |  |
| 30≦Age<40 (vs. 0≦Age<30) | 1.05 | (0.39-2.77) | 0.928 |  | 0.85 | (0.31-2.30) | 0.7468 |
| 40≦Age (vs. 0≦Age<30) | 6.68 | (2.90-15.38) | ***<0.001*** |  | 5.88 | (2.35-14.75) | ***<0.001*** |
| **Female (vs. male)** | 2.04 | (0.26-15.96) | 0.496 |  | 2.14 | (0.34-13.47) | 0.419 |
| **CHM use (vs. non-CHM use)** | 0.13 | (0.03-0.49) | ***0.003*** |  | 0.10 | (0.03-0.39) | ***<0.001*** |
| **Charlson comorbidity number_1-2 (vs. 0)** | 2.96 | (1.41-6.18) | ***0.004*** |  | 1.79 | (0.82-3.94) | 0.146 |
| **Charlson comorbidity number_≥3 (vs. 0)** | 1.90 | (0.34-10.52) | 0.462 |  | 0.92 | (0.14-6.16) | 0.934 |
| CNS, central nervous system; HR, hazard ratio; aHR, adjusted hazard ratio; CI, confidence interval; CHM, Chinese herbal medicine. | | | | | | | |
| Adjusted factors: age, sex, CHM use, and Charlson comorbidity number. | | | | | | | |
| The date after the 14 days’ cumulative prescription of CHM was designated the index date. | | | | | | | |
| Significant *p*-values (*p* < 0.05) are highlighted in bold italic font. | | | | | | | |
